# Microbial landscapes in dairy cow diseases: from localized dysbiosis to inter-organ axes

**DOI:** 10.1038/s41522-026-00988-8

**Published:** 2026-04-17

**Authors:** Pei Zhong, Ao Ren, Junwei Cui, Cheng Guo, Yanmei Zhang, Qiyu Diao, Xianjun Liu, Naifeng Zhang, Yan Tu, Yanliang Bi

**Affiliations:** 1 Institute of Feed Research, Chinese Academy of Agricultural Sciences, Key Laboratory of Feed Biotechnology of the Ministry of Agriculture and Rural Affairs, Beijing, China; 2 College of Animal Science and Veterinary Medicine, Shenyang Agricultural University, Shenyang, China; 3 College of Animal Science and Technology, Ningxia University, Yinchuan, China

**Keywords:** Biotechnology, Microbiology

## Abstract

Dairy cow health involves host-microbiome interactions. This review characterized microbial landscapes across anatomical sites in dairy cows—including the gastrointestinal tract, respiratory system, reproductive tract, mammary gland, and skin—and examines their associations with diseases. We elucidated how site-specific dysbiosis drives systemic conditions such as mastitis and ketosis through inter-organ axes. Finally, we evaluated emerging microbiome-based modulation strategies and their application prospects in dairy farming.

## Introduction

**Fig. 1:**
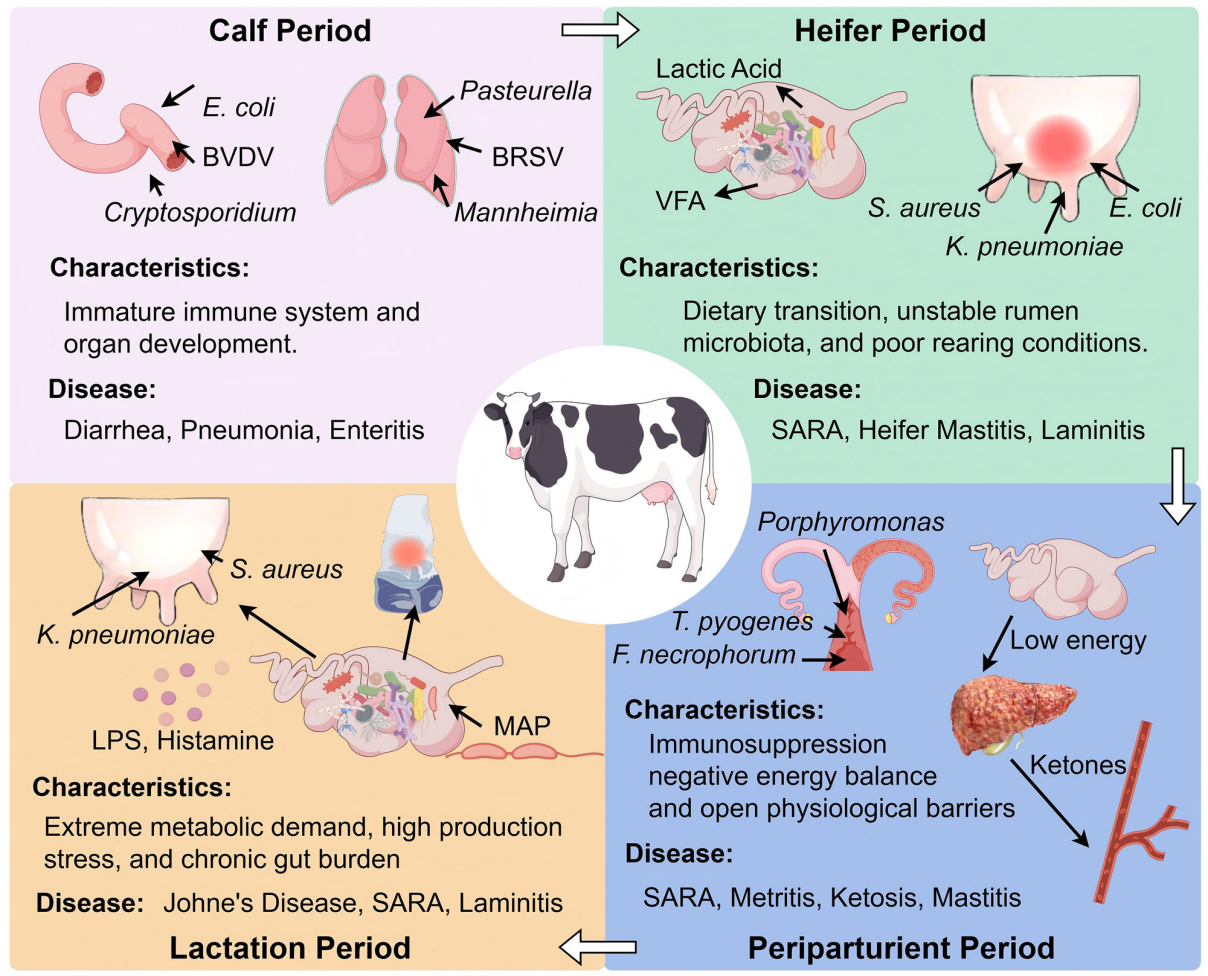
Major microbiome-associated diseases across the four life stages of dairy cows. The lifecycle is divided into four distinct phases: the Calf Period, characterized by an immature immune system and rapid organ development; the Heifer Period, marked by dietary transitions and reproductive maturation; the Periparturient Period, a high-risk transition phase involving severe metabolic stress and immunosuppression; and the Lactation Period, defined by extreme metabolic demands and chronic physiological burdens. Each stage highlights the key commensal and pathogenic microorganisms (e.g., *E. coli*, BRSV, *S. aureus*), critical metabolites (e.g., VFAs, LPS, Ketones), and the associated clinical or subclinical disorders (e.g., BRD, SARA, Metritis, and Mastitis) that arise from microbial-host dysbiosis. White arrows indicate the temporal progression of the production cycle. *E. coli* *Escherichia coli*, BVDV Bovine Viral Diarrhea Virus, BRSV Bovine Respiratory Syncytial Virus, VFA Volatile Fatty Acids, *S. aureus* *Staphylococcus aureus*, *K. pneumoniae* *Klebsiella pneumoniae*, *T. pyogenes* *Trueperella pyogenes*, *F. necrophorum* *Fusobacterium necrophorum*, LPS Lipopolysaccharide, MAP *Mycobacterium avium* subspecies *paratuberculosis*, SARA Subacute Ruminal Acidosis.

Dairy cows are central components of the global dairy supply chain, and their health and productivity are linked to food safety, animal welfare, and economic returns. As typical ruminants, dairy cows rely on microorganisms to support many physiological processes. Beyond the rumen, microbial communities inhabiting different segments of the gastrointestinal tract, as well as the respiratory tract, reproductive tract, mammary gland, and skin, collectively interact with the host to maintain homeostasis. However, dairy farming has long been constrained by microbe-associated diseases, spanning metabolic disorders, respiratory infections, reproductive failures, and mastitis^1^ (Fig. 1). Optimizing dairy cow health management from a microbial perspective therefore holds substantial promise.

With the rapid development of sequencing technologies, large-scale metagenomic datasets have revealed that site-specific microbiota perform distinct biological functions within defined microenvironments^2–5^, highlighting the heterogeneity of microbial composition across anatomical sites and underscoring the need for a multi-organ perspective. Conceptually, the dairy cow and its associated microbiota can be understood as a unified holobiont—a composite entity in which host and microbes co-regulate physiological homeostasis across multiple compartments^6^. Within this perspective, health depends not on a single organ, but on the coordinated interactions among networks throughout the organism. Studies have examined the rumen, uterine, or mammary microbiome separately, adopting a single-organ framework to elucidate disease etiology. However, increasing evidence indicates that distinct anatomical sites are functionally interconnected through systemic axes—such as the gut–mammary and gut–lung axes—mediated by microbial metabolites and immune signaling via circulation^7,8^. This inter-organ crosstalk provides new insights into the maintenance of health homeostasis and the development of systemic dysbiosis under disease conditions.

Given the critical role of the microbiota in health maintenance, the potential to reshape microbial communities through targeted modulation has received increasing attention. Current modulation strategies range from conventional approaches, such as antibiotics, probiotics, prebiotics, and synbiotics, to more advanced techniques, including antimicrobial peptides (AMPs), fecal microbiota transplantation (FMT), rumen microbiota transplantation (RMT), phage therapy, synthetic microbial communities (SynComs), and genetically engineered microorganisms (GEMs)^9–16^. Although these strategies have shown substantial progress in model organisms, their application within dairy cow farming remains at an early stage.

This review aims to map the microbial landscapes across key anatomical sites in dairy cows, elucidate the mechanistic links between localized dysbiosis and systemic diseases through inter-organ axes, and evaluate emerging microbiome-based intervention strategies. By integrating evidence across organ systems, we aim to provide a theoretical framework for understanding dairy cow health as an emergent property of the holobiont—offering a novel perspective for precision livestock management.

## The dairy cow microbiome: composition and health associations

Interactions between the host and its microbiota jointly shape health homeostasis, yet the pathogenic mechanisms arising from microbial imbalance across anatomical sites remain incompletely understood. This section, therefore, examines the compositional characteristics of the site-specific microbiome and its associations with disease.

### Oral microbiome and health associations in dairy cows

The oral cavity of dairy cows has been shown to harbor highly diverse microbial communities, mainly distributed across ecological niches such as the buccal surface, oral mucosa, and dental plaque^17–19^. Metagenomic data revealed a bacteria-dominated landscape (>90%), complemented by a complex array of anaerobic fungi, ciliates, and various viruses (Table 1).

**Table 1:** Documented dairy cow microbiome across various anatomical niches

Site	Prokaryotic microorganisms	Eukaryotic microorganisms	Viruses
Oral	Bacteria^17–20,34^:	Fungi^17,210^:	Eukaryotic Viruses^17,25–29,211,212^:
	Prevotellaceae, Lachnospiraceae, Ruminococcaceae, Lactobacillaceae, Succinivibrionaceae, etc.; Fusobacteriaceae, Porphyromonadaceae, Pasteurellaceae, Moraxellaceae, Neisseriaceae, etc.	Ciliophora, Chytridiomycota, Neocallimastigomycota	Nucleocytoviricota, BAdV, BRAV/BRBV, BCoV, VSV, FMDV, etc.
	Archaea^17^:	Protozoa^17,210^:	Phages^17^:
	Methanobacteria	*Entodinium*, *Diplodinium*, etc.	Uroviricota
Forestomach (Rumen)	Bacteria^67,213–215^:	Fungi^213,216–218^:	Eukaryotic Viruses^219^:
	Prevotellaceae, Lachnospiraceae, Ruminococcaceae, Bacteroidaceae, Oscillospiraceae, etc.	Neocallimastigomycota; Ascomycota & Basidiomycota (maybe Passenger), etc.	Partitiviridae, Alphaflexiviridae, Betaflexiviridae (ovine study), etc.
	Archaea^220,221^:	Protozoa^216^:	Phages^222,223^:
	Methanobacteriaceae, Methanomethylophilaceae, Methanomicrobiaceae, etc.	Ophryoscolecinae, Isotrichidae, Dasytrichidae, etc.	Caudoviricetes, Microviridae, Leviviricetes, etc.
Abomasum	Bacteria^66,67,224^:	Fungi^225^:	Eukaryotic Viruses^2^:
	Muribaculaceae, Ruminococcaceae, Oscillospiraceae, Lachnospiraceae, Sphaerochaetaceae, etc.; *Helicobacter canadensis*, *Acetobacter tropicalis*, *Mycoplasma* sp., etc.	*Rhizopus microsporus*, etc.	Megaviricetes, Pokkesviricetes, etc.
	Archaea^67^:	Protozoa:	Phages^2^:
	Methanomethylophilaceae, Methanocorpusculaceae, Methanobacteriaceae, etc.	Data limited	Caudoviricetes, Malgrandaviricetes, etc.
Small intestine	Bacteria^67,73,75^:	Fungi^73^:	Eukaryotic Viruses^82^:
	Clostridiaceae, Peptostreptococcaceae, Turicibacteraceae, Lachnospiraceae, Prevotellaceae, etc.	Neocallimastigomycota, etc.	BVDV, BCoV, etc.
	Archaea^73^:	Protozoa^73^:	Phages^2^:
	*Methanobrevibacter*, etc.	*Trichuris* (Parasites), etc.	Caudoviricetes, etc.
Large intestine	Bacteria^67,73^:	Fungi^73^:	Eukaryotic Viruses^2^:
	Oscillospiraceae, Bacteroidaceae, Clostridiaceae, Prevotellaceae, etc.	Neocallimastigomycota, etc.	Megaviricetes, Naldaviricetes, etc.
	Archaea^73^:	Protozoa^73^:	Phages^2^:
	*Methanobrevibacter*, *Methanocorpusculum*, etc.	*Trichuris* (Parasites), etc.	Caudoviricetes, etc.
Upper respiratory tract	Bacteria^103,104^:	Fungi^226^:	Eukaryotic Viruses^194,227^:
	Pasteurellaceae, Mycoplasmataceae, Moraxellaceae, Pseudomonadaceae, Enterobacteriaceae, etc.	*Aspergillus* spp., *Coccidioides immitis*, *Histoplasma capsulatum*, etc.	BRSV, BCoV, BPIV-3, BoHV-1, etc.
	Archaea:	Protozoa:	Phages:
	Data limited	Data limited	Data limited
Lower respiratory tract	Bacteria^8,110–112^:	Fungi:	Eukaryotic Viruses^194,228^:
	Pasteurellaceae, Mycoplasmataceae, Flavobacteriaceae, Clostridiaceae, Prevotellaceae, etc.	Data limited	BRSV, BVDV, BCoV, BPIV-3, BoHV-1, etc.
	Archaea:	Protozoa:	Phages:
	Data limited	Data limited	Data limited
Vagina	Bacteria^117,229,230^:	Fungi^231^:	Eukaryotic Viruses^232–234^:
	Mycoplasmataceae, Streptococcaceae, Corynebacteriaceae, Moraxellaceae, Fusobacteriaceae, etc.	Yeast, *Penicillium*, *Candida albicans*, *Aspergillus*, etc.	BoHV, Parapoxvirus, BLV, etc.
	Archaea^235^:	Protozoa:	Phages:
	*Methanobrevibacter*, etc.	Data limited	Data limited
Uterine and Cervical	Bacteria^124,125^:	Fungi^126^:	Eukaryotic Viruses^127^:
	*Escherichia*, *Mycoplasma*, *Ureaplasma*, *Serratia*, *Ruminococcus*, etc.	*Aspergillus* spp., *Penicillium* spp. etc.	BTV, etc.
	Archaea:	Protozoa:	Phages:
	Data limited	Data limited	Data limited
Udder	Bacteria^131–133,187^:	Fungi:	Eukaryotic Viruses:
	Non-*aureus* *Staphylococci*, *Corynebacterium*, *Staphylococcus aureus*, *Escherichia coli*, *Klebsiella* sp., Clostridiaceae, *Acinetobacter*, etc.	Data limited	Data limited
	Archaea:	Protozoa:	Phages^203,204^:
	Data limited	Data limited	Phages of *Staphylococcus* or *Escherichia coli*, etc.
Skin	Bacteria^137,138,140^:	Fungi^139^:	Eukaryotic Viruses^141^:
	*Treponema* spp., *Streptococcus pyogenes*, *Porphyromonas*, *Fusobacterium*, *Dermatophilus congolensis*, etc.	*Trichophyton verrucosum*, etc.	LSDV
	Archaea:	Protozoa:	Phages:
	Data limited	Data limited	Data limited

*BAdV* Bovine Adenovirus, *BRAV/BRBV* Bovine Rhinitis A Virus/Bovine Rhinitis B Virus, *BCoV* Bovine Coronavirus, *VSV* Vesicular Stomatitis Virus, *FMDV* Foot-and-Mouth Disease Virus, *BRSV* Bovine Respiratory Syncytial Virus, *BPIV-3* Bovine Parainfluenza Virus Type 3, *BoHV-1* Bovine Herpesvirus 1, *BLV* Bovine Leukemia Virus, *BTV* Bluetongue Virus, *LSDV* Lumpy Skin Disease Virus, *BVDV* Bovine Viral Diarrhea Virus.

The oral cavity is anatomically continuous with the forestomach, and microbial exchange occurs via rumination and saliva flow. This physiological connection results in an overlap in microbial composition between the oral cavity and the rumen^17,20^. Oral research showed that microbial diversity and the abundance of specific ASVs were associated with days in milk, milk yield, and residual feed intake, suggesting that the oral microbiome may serve as an indicator of nutritional status^21^. Consequently, researchers have also explored the potential of the oral microbiota as a predictive tool for ruminant diseases. For instance, a recent study demonstrated that subacute ruminal acidosis (SARA) significantly reshapes the oral microbiota and reduces its diversity. Specifically, the synchronous decline of Prevotellaceae_UCG-003 in both oral and ruminal sites highlights the potential of the oral microbiota as a non-invasive biomarker for SARA^22^.

In addition to serving as a biomarker, the oral microbiota also directly influences local tissue integrity and disease susceptibility. In dairy cattle, stomatitis is a common oral disease characterized by reduced feed intake, impaired mastication, excessive salivation, and erythema of the oral mucosa. Mechanical injuries caused by coarse forage awns or sharp seeds can induce local ulcers and inflammation^23^. However, infectious stomatitis often results in more severe clinical symptoms and production losses because of its contagious nature within the herd. For example, *Fusobacterium necrophorum* can induce necrotizing stomatitis through the secretion of toxins and proteolytic enzymes, resulting in fever, severe ulceration, and marked reductions in feed intake; in severe cases, lesions may extend to the pharynx^24^. In addition, several viruses can cause oral lesions. Vesicular stomatitis virus (VSV) induces vesicular and erosive lesions that reduce feed intake and milk yield^25–27^, whereas foot-and-mouth disease virus (FMDV) causes contagious vesicular lesions, often accompanied by hoof inflammation and mastitis, severely compromising feed intake and herd health^28,29^.

Beyond stomatitis, dental disorders in dairy cows also warrant attention. A previous study reported that approximately 28.3% of culled dairy cows exhibited dental abnormalities, with caries and periodontitis being the most common conditions^30^. Caries are typically caused by acid-producing bacteria, such as *Streptococcus* and *Lactobacillus*, which ferment carbohydrates and create a low-pH environment within dental biofilms, leading to tooth demineralization^31,32^. In severe cases, bacteria can damage alveolar bone and periodontal ligaments and result in periodontitis and tooth loss. Cows with periodontitis show pronounced oral dysbiosis, characterized by increased abundances of *Prevotella*, *Fusobacterium*, and *Porphyromonas* (Table 2). These taxa form a pathogenic synergistic network: *Prevotella* provides metabolic substrates through carbohydrate degradation, *Fusobacterium* enhances biofilm structural stability to facilitate persistent colonization, and *Porphyromonas* secretes virulence factors that damage periodontal tissues and suppress host immunity. The coordinated activity of these microorganisms is considered a driver of oral inflammation and progressive periodontal tissue degeneration^18,33,34^.

**Table 2:** Documented dysbiosis patterns and associated diseases across anatomical niches of dairy cows

Site	Disease	Dysbiosis characteristics	Clinical manifestations	References
Oral	Stomatitis	High loads of *Pseudomonas aeruginosa* and *Fusobacterium* spp. were identified	Inappetence, ataxia, and gastrointestinal ulceration	Tosaki et al.^24^
		Serological analysis revealed VSV-positive results	Lameness, vesicular and exudative lesions, and oral vesicles	Cargnelutti et al.^27^
		High copy numbers of FMDV RNA were detected in nasal secretions and blood samples	Oral vesicular lesions, viremia, and foot lesions	Arzt et al.^29^
		FMDV RNA was detected across multiple matrices	Fever, hypersalivation, vesicular stomatitis, interdigital dermatitis, and vulvitis	Suchowski et al.^28^
	Periodontitis	*Fusobacteria*, *Wolinella*, *Porphyromonas*, *Prevotella*, and *Treponema* exhibited high prevalence in clinical cases	Gingival recession, periodontal pockets, and purulent discharge	Borsanelli et al.^18^
		↑ *Caviibacter*, *Fusobacterium necrophorum*, *Moraxella* & *Mannheimia* in periodontitis	Gingival recession, periodontal pockets, and purulent discharge in calves	Vaccari et al^34^.
Forestomach (Rumen)	SARA	↓ Cellulolytic protozoa & G- bacteria; ↑ G+ bacteria; minimal effect on archaea & fungi	↓ DMI and milk yield	Elmhadi et al.^50^
		↑ *Prevotella* spp. *Succinivibrio dextrisolvens*, *Megasphaera elsdenii*, *Selenomonas ruminantium*; ↓ *Streptococcus bovis*	—	Plaizier et al.^236^
		↑ Succinivibrionaceae; ↓ Ruminococcaceae & Fibrobacteraceae	No significant change in DMI	Biber et al.^237^
		↑ *Stenotrophomonas* & *Succinivibrio*	Induction of mastitis	Hu et al.^178^
		↑ *Lactobacillus*, Streptococcaceae & *Bifidobacterium*; ↓ Bacteroidales, Lachnospiraceae, Clostridiaceae & Ruminococcaceae	—	Plaizier et al.^238^
	Ketosis	↓ *Ruminococcus* & *Prevotella*; ↓ propionate fermentation pathway	No significant change in milk yield	Kong et al.^47^
		Prevotellaceae & Ruminococcaceae negatively correlated with blood BHB; *Methanobrevibacter* positively correlated with BHB	—	Gebreyesus et al.^239^
		↓ *Ruminococcus*_*E bovis*; down-regulated AA metabolism, secondary metabolite biosynthesis & energy metabolism	↑ Milk fat & ↓ milk protein	Kong et al.^59^
		↑ *Mycoplasma* & *Saccharimonas*; ↓ *Limosilactobacillus*, *Lactobacillus*, *Ligilactobacillus* & *Bifidobacterium*	Abnormal rumen epithelium: slight thinning & tissue loosening	Li et al.^168^
		↓ *Prevotella* 7, *Ruminococcus* 2, *Selenomonas*, Veillonellaceae & Succinivibrionaceae	—	Wang et al.^56^
	Obese	↑ *Tidjanibacter* *inops_A*, *Rikenella massiliensis*, *Papillibacter cinnamivorans* & *Parabacteroides merdae*	Higher BCS & BWs; similar DMI	Li et al.^61^
	Milk fat depression	↑ *Prevotella*; ↓ Lachnospiraceae, *Oribacterium*, Veillonellaceae & *Pseudobutyrivibrio*	↓ Milk fat % & fat yield; ↑ lactose	Zeng et al.^63^
		↓ Fungi & ciliated protozoa; ↑ *Streptococcus bovis*	↓ Milk fat yield	Rico et al.^62^
Abomasum	Abomasitis	Abnormal expansion of *Clostridium perfringens* (types A/C/D), *Sarcina*, *Salmonella enterica*, and *Clostridium septicum*	Gastric hemorrhage or perforation, leading to peritonitis	Bus et al.^68^
Small intestine	Paratuberculosis	Caused by *Mycobacterium avium* subsp. *paratuberculosis* infection	Chronic diarrhea, weight loss, and reduced milk production	Griss et al.^240^
	Hemorrhagic bowel syndrome	Abnormal expansion of *Clostridium perfringens* type A	↓ DMI & milk yield; rapid emaciation; abdominal bloat; melena or bloody feces	Elhanafy et al.^79^
	Diarrhea	Caused by BVDV, BCoV, *Escherichia coli*, *Clostridium perfringens* & *Cryptosporidium* spp.	Inappetence; watery diarrhea; potential mortality	Jessop et al.^82^
Large intestine	Hindgut acidosis	↓ *Fibrobacter*, *Ruminococcus*, *Ruminiclostridium*, *Methanobrevibacter*, *Methanosarcina* & *Methanosphaera*	—	Zhang et al.^241^
		↑ *Bifidobacterium*, *Acetitomaculum* & *Butyrivibrio*	—	Rivera-Chacon et al.^92^
Respiratory system	Bovine Respiratory Disease	*Pasteurella multocida*, *Histophilus somni* & *Mycoplasma bovis* associated with pneumonia	Cough; RR > 40 bpm; cranio-ventral lung sounds or wheezing	Gaeta et al.^103^
		*↑* Nasopharyngeal *Pasteurella spp*.	Ultrasonographic diagnosis of lobar pneumonia	Raabis et al.^105^
		High loads of *Pasteurella multocida* & *Mycoplasma bovis* in tracheobronchial lavage	Cough; RR > 40 bpm; rectal hyperthermia	Kaura et al.^97^
		*Trueperella pyogenes*, *Pasteurella multocida*, *Histophilus somni*, *Mycoplasma* spp., BoHV-1, BRSV, BVDV & BPIV-3 detected in lung	Mortality due to pneumonia	Zhou et al.^228^
Reproductive system	Postpartum diseases	Proteobacteria, Fusobacteria & Bacteroidetes associated with RFM	Failure to expel fetal membranes within 24 h postpartum	Bicalho et al.^117^
		Enrichment of *Trueperella pyogenes*, *Fusobacterium necrophorum* & *Porphyromonas levii*	Purulent vaginal discharge	Moore et al.^118^
		Outbreak of bovine necrotic vulvovaginitis caused by pathogenic *Porphyromonas levii*	Clitoral/vaginal mucosal lesions, necrotic tissue & vulvar swelling	Elad et al.^122^
		Mixed infection of BoHV-1, *Ureaplasma diversum* & *Mycoplasma bovigenitalium*	Infectious pustular vulvovaginitis	Connella et al.^120^
		*Mycoplasma bovigenitalium* infection	Bovine necrotic vulvovaginitis	Lysnyansky et al.^121^
Udder	Mastitis	Expansion of *Staphylococcus aureus*	↑ Somatic cell count; abnormal changes in milk & mammary tissue	Dego et al.^131^
		Expansion of *Escherichia coli* & *Klebsiella* spp. under high heat & humidity	Mastitis	Gao et al.^132^
		*Klebsiella pneumoniae* K57 (capsular serotype) as the primary mastitis strain	Mastitis	Yang et al.^134^
Skin (including hoof)	Digital Dermatitis	Synergistic pathogenesis of *Treponema* spp., *Porphyromonas* & *Fusobacterium*	Lameness	Caddey et al.^138^
	Udder cleft dermatitis	*Streptococcus pyogenes* virulence factors hindering skin recovery	Dysregulated inflammation; impaired skin healing & barrier dysfunction	Vermeersch et al.^142^
	Dermatitis	*Trichophyton verrucosum* infection	Focal alopecia & crusty dermatitis	Ming et al.^139^
		*Dermatophilus congolensis* infection	Skin lesions	Lagneau et al.^140^

*SARA* Subacute Ruminal Acidosis, *DMI* Dry Matter Intake, *BCS* Body Condition Score, *BW* Body Weight, *BHB* β-hydroxybutyrate, *AA* Amino Acid, *VSV* Vesicular Stomatitis Virus, *FMDV* Foot-and-Mouth Disease Virus, *BVDV* Bovine Viral Diarrhea Virus, *BCoV* Bovine Coronavirus, *BoHV-1* Bovine Herpesvirus Type 1, *BRSV* Bovine Respiratory Syncytial Virus, *BPIV-3* Bovine Parainfluenza Virus Type 3, *RFM* Retained Fetal Membranes, *RR* Respiratory Rate, *G* *+* Gram-positive, *G−* Gram-negative.

### Forestomach microbiome and metabolic function

As the central hub of fermentation, the rumen accounts for approximately four-fifths of the forestomach volume^35^. It hosts an exceptionally dense microbial population (10^11^ cells/mL), dominated by bacteria but also including archaea, protozoa, and anaerobic fungi, a composition distinct from monogastric systems (Table 1).

Rumen physiological function depends on complex multi-domain microbial interactions. For example, in fiber degradation, anaerobic fungi develop rhizoids that penetrate plant cell walls and secrete cellulosomes to disrupt fiber, increasing bacterial accessibility^36,37^. Bacterial communities then show substrate-specific functional partitioning: dominant taxa such as *Butyrivibrio*, Lachnospiraceae, and *Ruminococcus* perform initial breakdown of lignocellulose; *Streptococcus*, *Enterococcus*, and *Clostridium* further metabolize simple substrates into volatile fatty acids (VFAs), ethanol, and hydrogen, which methanogens ultimately convert to methane^38^. Protozoa secrete active cellulases and remove free oxygen, maintaining anaerobic conditions^39^. Recent studies also show that phages modulate community structure via host lysis, affecting feed degradation and fermentation^40^. Conversely, rumen bacteria have also evolved an antiviral arsenal, including restriction-modification and CRISPR-Cas systems, to collectively defend against phage attacks^41^. Collectively, this functionally complementary microbial network forms the fundamental basis of rumen homeostasis.

Beyond substrate degradation, hydrogen metabolism and methanogenesis exemplify the tight cross-kingdom interactions that sustain rumen homeostasis. During anaerobic fermentation, bacteria, anaerobic fungi, and protozoa continuously generate hydrogen as an electron sink to maintain redox balance. Most hydrogen-producing bacteria rely on [FeFe]-hydrogenases^42^, while some anaerobic fungi and protozoa utilize specialized hydrogenosomes for hydrogen release^43,44^. Accumulation of hydrogen elevates intraruminal hydrogen partial pressure, which inhibits fiber fermentation and suppresses VFA production^45^. Rumen archaea, predominantly *Methanobrevibacter*, convert hydrogen and carbon dioxide to methane, thereby maintaining the low hydrogen partial pressure required for efficient fermentation, but simultaneously producing the greenhouse gas methane^45^. Although archaea are not classical pathogens, disruption of archaeal populations have been associated with this interspecies network, contributing to metabolic imbalance^46^. Emerging evidence indicates that archaeal diversity and community composition are altered in metabolic disorders such as ketosis and SARA^47,48^.

Disruption of the rumen microbial network can shift metabolism into pathological states. SARA is characterized by a drop in pH due to VFA and lactate accumulation. This dysbiosis is characterized by the proliferation of acid-producing bacteria and the decline of fiber-degrading bacteria (Table 2), ultimately triggering inflammation and impairing production performance^49,50^. RMT from healthy donors partial restored rumen microbial homeostasis and mitigated SARA, providing evidence for the causal involvement of microbes in SARA development^51^. During SARA, rapid microbial fermentation leads to the accumulation of lipopolysaccharide (LPS) and histamine, which downregulate tight junction proteins and increase epithelial permeability, thereby compromising the structural integrity of the ruminal epithelial barrier^52^. These localized microbial and metabolic alterations induce inflammatory responses within the ruminal mucosa, the systemic consequences of which will be further addressed in the inter-organ axes section^53,54^.

The rapid dietary shift from high-fiber to high-concentrate feeds during the periparturient period profoundly reshapes the rumen microbial landscape. Dysbiosis during this transition underlies elevated ketosis risk: the decline of VFA-producing taxa—including *Ruminococcus*, *Prevotella*, *Selenomonas*, and Succinivibrionaceae—impairs propionate fermentation pathways and reduces gluconeogenic precursor supply, while the concurrent expansion of opportunistic taxa such as *Mycoplasma* and *Saccharimonas* further destabilizes ruminal homeostasis (Table 2), collectively contributing to hypoglycemia, excessive mobilization of body fat, and ketone accumulation, which compromise lactation, reproductive performance, and offspring health^47,55–57^. The causal role of the microbiome in ketosis pathogenesis is supported by studies where RMT from healthy donors accelerated microbial re-establishment and stabilized fermentation, thereby significantly suppressing plasma β-hydroxybutyrate (BHBA) levels^58^. Strain isolation and cell-based studies indicate that *Ruminococcus_E bovis*, which is significantly downregulated in the rumen of ketotic cows, can provide gluconeogenic precursors such as alanine, thereby promoting hepatic glucose production and reducing ketone formation^59^.

Rumen dysbiosis is also linked to other metabolic disorders. Fatty liver is associated with rumen dysbiosis-induced negative energy balance and altered microbial metabolite profiles that exacerbate hepatic lipid accumulation^60,61^; milk fat depression correlates with abnormal carbohydrate-degrading bacterial structures^62,63^; and rumen bloat relates to activity changes in gas-producing and acid-producing bacteria, such as *Streptococcus bovis* and *Succinivibrio*^64,65^. Despite differing pathologies, these conditions share a common feature: rumen microbial imbalance and altered metabolic function.

### Abomasal microbiome and abomasitis

Studies on the abomasal microbiome remain limited, mainly due to the difficulty of sample collection, which often requires slaughter. Compared with the forestomach, the abomasal microbiome exhibits significantly lower diversity, a characteristic dictated by the transition to a high-acid environment. Metagenomic analysis revealed that while the abomasal contents harbor specific bacterial and archaeal families (Table 1), the community structure differs markedly from that of the rumen, reflecting a shift toward acid-tolerant populations^66,67^. The viral landscape was similarly specialized, dominated by distinct classes of phages and eukaryotic viruses. Beyond its primary roles in hydrochloric acid secretion and pepsinogen-mediated protein digestion, this niche also functions as a biological filter that restricts microbial passage; yet its lower microbial complexity renders it a distinct site for potential dysbiosis, particularly during the early stages of host development^68^.

Currently, research on the functions of the healthy abomasal microbiome is limited, but evidence suggests that microbial dysbiosis in the abomasum is closely associated with disease (Table 2). Neonatal calves, with immature immunity and an underdeveloped gastrointestinal system, are susceptible to abomasitis, primarily caused by toxigenic bacteria such as *Clostridium perfringens* types A/C/D, *Sarcina*, *Salmonella enterica*, and *Clostridium septicum*^68,69^. Pathogen proliferation drives the secretion of a toxin cocktail (α-toxin, perfringolysin O, and β2-toxin) that disrupts the abomasal mucosal barrier, which triggers necrosis, hemorrhage, and intramural gas accumulation and ultimately leads to severe structural degradation of the abomasal wall^70,71^. Clinically, these lesions commonly manifest as anorexia, acute bloat, and diarrhea. In severe cases, bacterial translocation and systemic inflammatory spread can lead to mortality rates as high as 50%^72^. While bacterial pathogens are primary drivers, abomasal homeostasis is further challenged by a diverse array of non-bacterial agents. Opportunistic fungi (*Saksenaea erythrospora*), parasites (*Ostertagia ostertagi*), and viruses (Bovine viral diarrhea virus) can independently or synergistically induce abomasitis, contributing to systemic dissemination of infection^68^.

### Small intestinal microbiome and susceptibility to enteric disorders

Characterizing the small intestinal microbiome in dairy cows is challenging due to the difficulty of in vivo sampling and low microbial density. Metagenomic data revealed a bacterial landscape dominated by families such as Clostridiaceae and Peptostreptococcaceae, with *Methanobrevibacter* representing the primary archaeal component (Table 1). Interestingly, the relatively higher abundance of polysaccharide-degrading taxa such as *Prevotella* in the duodenum compared to the jejunum and ileum may reflect passive transfer from the rumen rather than indigenous colonization, highlighting the role of the small intestine as a transitional zone in the digestive continuum^73^.

Although low in abundance, small intestinal microbes contribute to host metabolic regulation. Metagenomic analysis indicated enrichment in oxidative phosphorylation, arachidonic acid, and tryptophan metabolic pathways, as well as numerous GH109 family enzymes involved in mucin degradation^73^. In vitro evidence demonstrated that propionate upregulated gluconeogenic rate-limiting enzymes—including phosphoenolpyruvate carboxykinase 2 (PEPCK2) and fructose-1,6-bisphosphatase 1 (FBP1)—within jejunal epithelial cells, thereby supporting local mucosal energetics^74^. Notably, insufficient energy supply to the mucosa is a critical predisposing factor for compromised barrier function, which can trigger intestinal inflammation and bacterial translocation. Furthermore, specific ileal taxa, notably *Paraclostridium*, actively participate in bile acids (BAs) metabolism via the secretion of bile salt hydrolase (BSH). This microbial deconjugation of BAs modulated the farnesoid X receptor (FXR)/fibroblast growth factor 19 (FGF19) signaling axis^75^. Disruption of this signaling axis was also closely associated with intestinal barrier impairment, inflammatory responses, and systemic metabolic disorders.

Small intestinal dysbiosis serves as a primary driver for the development of diverse enteric disorders. High-concentrate diets disrupt microbial homeostasis, leading to the intraluminal accumulation of LPS within the jejunum and ileum. This process triggers a potent inflammatory response, characterized by the upregulation of pro-inflammatory cytokines—including IL-1β and interferon-γ (IFN-γ)—and the concomitant downregulation of tight junction proteins, thereby compromising mucosal barrier integrity^76^. In addition to nutritional factors, pathogenic infections also drive dysbiosis. Paratuberculosis, caused by *Mycobacterium avium* subsp. *paratuberculosis* (MAP), primarily invades the distal ileum via M cells, persists in macrophages through intracellular immune evasion, and results in granuloma formation, villus atrophy, mucosal thickening, and barrier dysfunction, ultimately leading to chronic diarrhea, weight loss, and reduced milk production^1,77,78^. In high-yielding dairy cows, sporadic hemorrhagic bowel syndrome (HBS) is associated with very high mortality (>85%), likely linked to the combined effects of *Clostridium perfringens* type A and fumonisins^79–81^. In calves, small intestinal microbiota is less stable, resulting in higher disease incidence, including neonatal calf diarrhea (caused by Bovine viral diarrhea virus, Bovine coronavirus (BCoV), enterotoxigenic *Escherichia coli*, *Clostridium perfringens*, and *Cryptosporidium* spp.) and necrotizing enterocolitis^82^.

### Large intestinal microbiome: secondary fermentation and metabolic function

The microbial composition of the cecum, colon, and rectum is generally similar. Due to the relative ease of sampling, the rectum is often used as a representative of hindgut microbiota^67^. Compared to the small intestine, the hindgut harbors a highly diverse microbiota (Table 1).

The large intestinal lumen, with its significantly greater cross-sectional area that slows chyme transit, provides sufficient time for secondary fermentation of residual nutrients by hindgut microbes, which in turn regulate host physiology via complex secondary metabolites^83^. For example, *Bifidobacterium pseudolongum*-mediated indole derivatives correlated positively with growth performance in calves^84^. In lactating cows, hindgut microbial-mediated secondary BAs metabolism was closely associated with plasma glucose and BHBA levels, mainly through remodeling taurine-conjugated and deconjugated BAs profiles to influence lipid absorption, suggesting the hindgut “microbiota–BAs axis” is critical for adapting to periparturient negative energy balance^85^. Additionally, hindgut-derived short-chain fatty acids (SCFAs), specifically acetate and propionate, activated the Nrf2–Keap1 pathway. This activation enhanced host antioxidant capacity and mitigated postpartum oxidative stress^86,87^.

Hindgut microbial stability is sensitive to diet, antibiotics, and other external factors, which can trigger systemic pathological responses. Antibiotic intervention significantly reduced hindgut microbial diversity, with slow recovery^88^. Nutritional diarrhea induced by abrupt postpartum diet changes is associated with decreased total SCFAs and increased ammonia, indicating impaired microbial fermentation^89^. Similar to the rumen, starch overflow induced hindgut acidosis, leading to LPS release by Gram-negative bacteria such as Enterobacteriaceae; the resulting mucosal injury and inflammation closely resemble mechanisms in acidosis^90–92^. Conversely, specific hindgut microbes can serve as targets to alleviate metabolic disorders. For example, citrus flavonoid supplementation remodeled microbial communities, increasing Bacteroidaceae abundance and upregulating sphingolipid metabolism, which enhanced mucosal barrier function and attenuated LPS-induced inflammatory signaling^93^. Researchers observed that dairy cows harboring a *Bifidobacterium*-dominated enterotype exhibited faster recovery following LPS challenge, with accelerated restoration of SCFAs, enhanced amino acid metabolism, improved glucose homeostasis, and higher milk fat and protein content compared to other enterotypes, suggesting that intrinsic microbiome configurations may determine host resilience to inflammatory challenges^94^.

### Upper respiratory tract microbiome: host immunity and pathogen dynamics

Recent multi-omics studies have redefined the respiratory tract as a microbial ecosystem with distinct spatial compartments and ecological gradients. As a critical biological component of the upper respiratory tract (URT) mucosal barrier, URT microbiota plays a fundamental role in maintaining local immune homeostasis and reinforcing barrier defenses against environmental pathogens^95,96^. The URT includes the nasal cavity, nasopharynx, oropharynx, and tonsils, with microbes primarily adhering to the mucosal surface rather than being suspended in the lumen^95^. Metagenomic data indicated that this niche is overwhelmingly dominated by specific bacterial families, particularly Pasteurellaceae and Mycoplasmataceae^97^, which constitute the core commensal landscape (Table 1). In contrast, viruses and fungi represent lower-abundance components; however, primary respiratory viruses and opportunistic fungi can infiltrate these compartments under environmental stress.

In production settings, immunocompromised cattle are highly susceptible to URT dysbiosis, which often leads to bovine respiratory disease (BRD)^98^, characterized by the overgrowth of opportunistic bacteria, including *Pasteurella multocida*, *Histophilus somni*, *Mannheimia haemolytica*, and *Mycoplasma bovis*, frequently in co-infection with respiratory viruses such as Bovine respiratory syncytial virus (BRSV), Bovine viral diarrhea virus (BVDV), and Bovine parainfluenza virus type 3 (BPIV-3) (Table 2). Synergistic interactions among pathogens exacerbate tissue damage through multiple mechanisms^99^. First, viral infections predisposed the respiratory epithelium to bacterial colonization by inducing the overexpression of host surface receptors, such as platelet-activating factor receptor (PAFR) and intercellular adhesion molecule 1 (ICAM-1). Specifically, pre-infection with BRSV or BCoV was shown to markedly enhance the adherence of *Pasteurella multocida*^100^. Second, viral-bacterial co-infections cooperatively compromised mucosal barrier integrity. For instance, the synergistic action of BRSV and *Histophilus somni* downregulated tight junction proteins and stimulated the aberrant secretion of matrix metalloproteinases (notably MMP1 and MMP3), thereby accelerating structural degradation and inflammatory infiltration^101^. Third, these polymicrobial interactions amplified pathological immune signaling. Co-infection with BRSV and *Mannheimia haemolytica* triggered an exacerbated IL-17 response, which drove dysregulated neutrophil recruitment and perpetuated extensive immunopathological injury to the lung parenchyma^102^.

It is noteworthy that these potential pathogens are also detectable in healthy individuals, indicating that BRD development is more associated with disruption of host microbial balance than with simple pathogen invasion^103^. For instance, early-onset pneumonia in calves showed increased total bacterial load and decreased α-diversity by day 3 after birth, suggesting that BRD arose from opportunistic pathogen overgrowth under dysbiotic conditions^104,105^. Notably, Malmuthuge et al. observed that the weaning-induced expansion of respiratory pathogens coincided with alterations in the expression of immune-related genes in blood leukocytes, suggesting that weaning-triggered endocrine shifts further compromised systemic immunological surveillance^106^. Environmental stressors further modulated susceptibility: high ammonia levels damaged mucosal barriers and impaired tight junctions^107^, while thermal stress activated the hypothalamic–pituitary–adrenal axis, inducing stress-related immunosuppression and weakening respiratory mucosal defense^108^. Collectively, BRD development reflects the combined influence of host immune status, environmental stress, and microbial interactions.

### Lower respiratory tract barriers and low-biomass microbiota

The lower respiratory tract (LRT), comprising the trachea, bronchi, and alveoli, harbors bacterial communities with significantly lower abundance and diversity compared to the URT^95,109^. Using invasive sampling methods, such as bronchoalveolar lavage (BAL) or transtracheal aspiration (TTA), the LRT has been shown to host a low-biomass core microbiota dominated by Pasteurellaceae, Mycoplasmataceae, Flavobacteriaceae, Clostridiaceae, and Prevotellaceae, while archaea, viruses, and eukaryotic microbes are rarely reported (Table 1)^8,110–112^.

Under healthy conditions, the bovine LRT maintains microbial homeostasis through multiple barrier systems. Physically, the mucus–ciliary clearance system continuously removes mucus-encased microbes toward the pharynx^113^. Chemically, surfactants secreted by type II alveolar cells and antimicrobial peptides produced by airway epithelial cells or neutrophils exert direct antimicrobial effects^114^. Immunologically, alveolar macrophages recognize pathogen-associated molecular patterns (PAMPs) via Toll-like receptors (TLRs) TLR2 and TLR4, rapidly phagocytose pathogens, and present antigens; secretory immunoglobulin A (IgA) blocks bacterial adhesion, while regulatory T cells suppress excessive inflammation^115^. Colonizing commensals, such as *Prevotella* and *Veillonella*, may produce SCFAs that lower local pH to inhibit pathogens and act as immune signaling molecules to enhance host defense^115^. Through the coordinated action of physical, chemical, immune, and microbial barriers, the LRT generally maintains a low-biomass, stable microbial ecosystem. However, when viral infection, environmental stress, or immunosuppression impairs these defenses, microbes from the oral cavity and URT can aberrantly colonize the LRT, markedly increasing the risk of BRD through pathogenic mechanisms and microbial agents similar to those described in the URT section above.

### Vaginal microbiome and reproductive health

The health of the bovine reproductive system directly affects reproductive efficiency and offspring health, representing a critical factor for sustainable dairy production. Consequently, the periparturient reproductive tract microbiota and its association with reproductive disorders have received increasing attention. The bovine vaginal microenvironment is characterized by a near-neutral pH (6.5–7.5) and scant glycogen availability, contrasting sharply with the acidic, *Lactobacillus*-dominant human vagina. In this niche, microbes utilize mucins and nitrogenous compounds as primary substrates, which precludes the dominance of lactic acid-producing bacteria^116^. Metagenomic and culture-based data indicated that this ecosystem is primarily colonized by specific bacterial families alongside lower-abundance populations of archaea, yeasts, and clinically relevant viruses (Table 1).

While the full ecological function of the bovine vaginal microbiota remains under investigation, evidence strongly links periparturient dysbiosis to reproductive disorders. In a longitudinal study spanning pre- and post-parturition, healthy individuals exhibited peak vaginal microbial diversity on the day of calving, whereas those developing metritis already showed significantly reduced diversity at parturition. Community dynamics further revealed that vaginal microbial load generally increased 3–7 days postpartum, with a more pronounced expansion in cows with retained fetal membranes (RFM). This process was primarily driven by the abnormal proliferation of Fusobacteriota and Bacteroidota: Bacteroidota could efficiently degrade host mucins and polysaccharides, providing essential carbon and energy sources for the community, while Fusobacteriota might exploit their secreted virulence factors, such as leukotoxins and extracellular proteases, to disrupt the mucosal structure^117^.

Vaginal dysbiosis can further induce complex inflammatory phenotypes. The high incidence of purulent vaginal discharge (PVD) in the first three weeks postpartum is significantly associated with early vaginal microbial features, characterized by enrichment of *Trueperella pyogenes*, *Fusobacterium necrophorum*, and *Porphyromonas levii*. Functional gene predictions suggest that these pathogens mediate persistent local inflammation by enhancing biofilm formation and compromising mucosal barrier function^118^. Periparturient physiological immunosuppression further weakens vaginal defenses, promoting abnormal colonization by opportunistic pathogens. On some farms, such dysbiosis can develop into bovine necrotic vulvovaginitis (BNVV), a polymicrobial infection not attributable to a single pathogen, but driven by bacteria such as *Porphyromonas*, *Mycoplasma*, *Ureaplasma*, and *Parvimonas*, alongside viruses including Bovine herpesvirus (BoHV) and Parapoxvirus. In affected tissues, multiple microbial populations invade the submucosa, accompanied by impaired neutrophil recruitment. The occurrence of this disease was significantly associated with reduced conception rates, extended calving intervals, and decreased milk production^119–122^.

### Uterine and cervical microbiome and reproductive disorders

As the ascending extension of the vagina, the cervix and uterus form the internal reproductive tract, connected via the cervical canal, and undergo substantial microbial remodeling during parturition. The uterus was historically considered sterile under healthy conditions; however, recent studies have detected microbial 16S rRNA signals in the cervical lumen, placenta, and even amniotic fluid of normally pregnant cows^123^. Opportunistic pathogens were present in both healthy and diseased uteri, but healthy individuals exhibited higher microbial heterogeneity and richness (Table 1). Core uterine bacteria were dominated by Proteobacteria (e.g., *Escherichia*, *Serratia*), Mycoplasmota (*Mycoplasma*, *Ureaplasma*), and Firmicutes (e.g., *Ruminococcus*), whereas archaea are rarely reported^124,125^. Among eukaryotes, *Aspergillus spp*. and *Penicillium spp*. have been detected in metritis uteri^126^. Viruses are infrequently reported in the uterus, although detection in neonates suggests potential in utero transmission; for example, placental infection with live-attenuated Bluetongue virus (BTV) led to congenital hydranencephaly in calves^127^.

Uterine microbial assembly may occur through a dynamic interplay of ascending migration from the lower reproductive tract and endogenous translocation via the systemic circulation. During the peripartum period, the dilation of the cervix facilitated the ascending entry of vaginal residents, including key pathogens such as *Trueperella pyogenes*, *Escherichia coli*, and *Fusobacterium necrophorum*^128^. Crucially, the hematogenous route has gained increasing recognition as a non-canonical pathway for uterine seeding. The detection of *Fusobacterium* and other anaerobic taxa in peripheral blood around parturition suggested that intestinal or oral barrier compromise might allow for the systemic dissemination of these microbes, which subsequently colonize the uterus^129^. Experimental infusion of defined pathogen consortia into the uterus has successfully reproduced endometritis phenotypes, providing direct evidence for a causal role of microbial imbalance in uterine pathology^130^. Although emerging evidence suggests that specific pathogens can colonise and exert pathological effects within the uterus, the mechanisms underlying maternal-fetal transmission and the downstream consequences for fetal health remain important directions for future investigation.

### Mammary microbiome and mastitis

Clinical and subclinical mastitis represents one of the most economically damaging challenges in dairy farming, causing approximately $22 billion (USD) in global annual losses^1^. *Staphylococcus aureus* is the most representative mastitis pathogen. It adheres to epithelial cells via fibronectin-binding proteins (FnBPs) and evades host immunity through biofilm formation and intracellular persistence. Its secreted hemolysins and exotoxins directly damage tissue and trigger inflammation^131^. Large-scale epidemiological surveys in Chinese commercial farms indicated that under conditions of high temperature—humidity or organic bedding, milk frequently tested positive for pathogens such as *Escherichia coli* and *Klebsiella* spp., which ascended via the teat canal, thereby shifting the etiological profile toward environmental pathogens^132^. Recent studies further clarified that *Klebsiella pneumoniae* rapidly invaded bovine mammary epithelial cells, causing mitochondrial damage and apoptosis while triggering a robust release of pro-inflammatory cytokines such as IL-6, IL-8, and TNF-α^133^. Notably, the *Klebsiella pneumoniae* K57 capsular serotype has been identified as a predominant lineage in bovine mastitis, exhibiting significant genetic diversity. Its pathogenicity is closely linked to specific adhesion-associated genes (e.g., fimA, sfaA, and focA) and aminoglycoside-resistance genes (aph(6)-Id, strAB), which facilitate both mucosal colonization and evasion of conventional antimicrobial therapy^134^.

Beyond exogenous infection, endogenous mechanisms mediated by the “gut–mammary axis” have recently gained attention. FMT experiments provided preliminary evidence for a causal link between gastrointestinal dysbiosis and mammary inflammation^135^, the detailed mechanisms of which will be comprehensively reviewed in the following section.

### Skin and hoof microbiota and associated infectious diseases

As the first physical barrier against the external environment, the microbiota of bovine skin and hooves is regulated by host genetics, mechanical damage, and environmental factors such as bedding hygiene and humidity.

In hoof health, digital dermatitis (DD) is a primary microbial trigger of lameness in dairy cows. DD is thought to result from prolonged exposure of the hooves to high-moisture, high-organic-load environments, leading to microbial dysbiosis. Studies have revealed that bacterial communities at lesions of both acute ulcerative and chronic proliferative DD differed significantly from those on healthy skin. These alterations are characterized by a marked increase in the relative abundance of Spirochaetota and Fusobacteriota, coupled with an overall reduction in bacterial diversity^136^. Moreover, DD is not caused by a single pathogen but by the synergistic action of multiple pathogenic bacteria. Among these, *Treponema* spp. have been identified as the core driver of disease progression; they utilize chemotaxis and motility to penetrate damaged keratin layers into deeper epidermal layers^137^. Subsequently, *Porphyromonas* and *Fusobacterium* exacerbated tissue necrosis by secreting proteases and keratin-degrading enzymes, ultimately inducing characteristic ulcerative lesions^138^.

The integrity of the skin barrier is critical for limiting opportunistic pathogen invasion. When the barrier is compromised by trauma, parasites, or environmental stress, the expansion of specific pathogens can lead to severe skin lesions. For example, *Trichophyton verrucosum* secreted keratinases that degraded hair and keratin, causing focal alopecia and crusted dermatitis. Under persistently wet conditions, zoospores of *Dermatophilus congolensis* penetrated damaged epidermis and formed branching filaments, inducing exudative and crusted dermatitis^139,140^. Viral infections can also impair barrier function by amplifying inflammatory responses. For instance, Lumpy skin disease virus (LSDV) encodes the protein LSDV001, which has been shown to enhance NF-κB pathway activation induced by IL-1β and TNF-α; this excessive inflammatory response not only worsens nodular lesions but also creates a window for secondary microbial invasion^141^. Udder skin is also susceptible to dysbiosis-driven disease. Udder cleft dermatitis (UCD), a multifactorial dermatological condition of the udder skin, is characterized by overgrowth of opportunistic bacteria and impaired skin barrier function. Multi-omics analyses have revealed that the virulence factors of the facultative pathogen *Streptococcus pyogenes* contributed to chronic dysregulated inflammation and hampered wound healing^142^. In summary, whether in hoof DD or other skin infections, the underlying process involves disruption of skin/hoof microbial homeostasis under external stress, leading to loss of protective microbial function and dominance of pathogenic microorganisms.

### From regional dysbiosis to inter-organ communication

The previous chapter systematically reviewed the diverse microbial communities colonizing different anatomical sites in dairy cows and their functional characteristics (Tables 1 and 2), highlighting the close associations between these microbes and host metabolic homeostasis, immune regulation, and disease development. While each anatomical niche harbors a specialized microbiota, localized dysbiosis and barrier failure can transition into systemic pathology through the translocation of microbial signals and metabolites. Viewing these compartments as an interconnected network—defined by inter-organ axes—is essential to understanding how the bovine holobiont drives complex disease progression.

## Mediators facilitating the inter-organ axes in systemic diseases

**Fig. 2:**
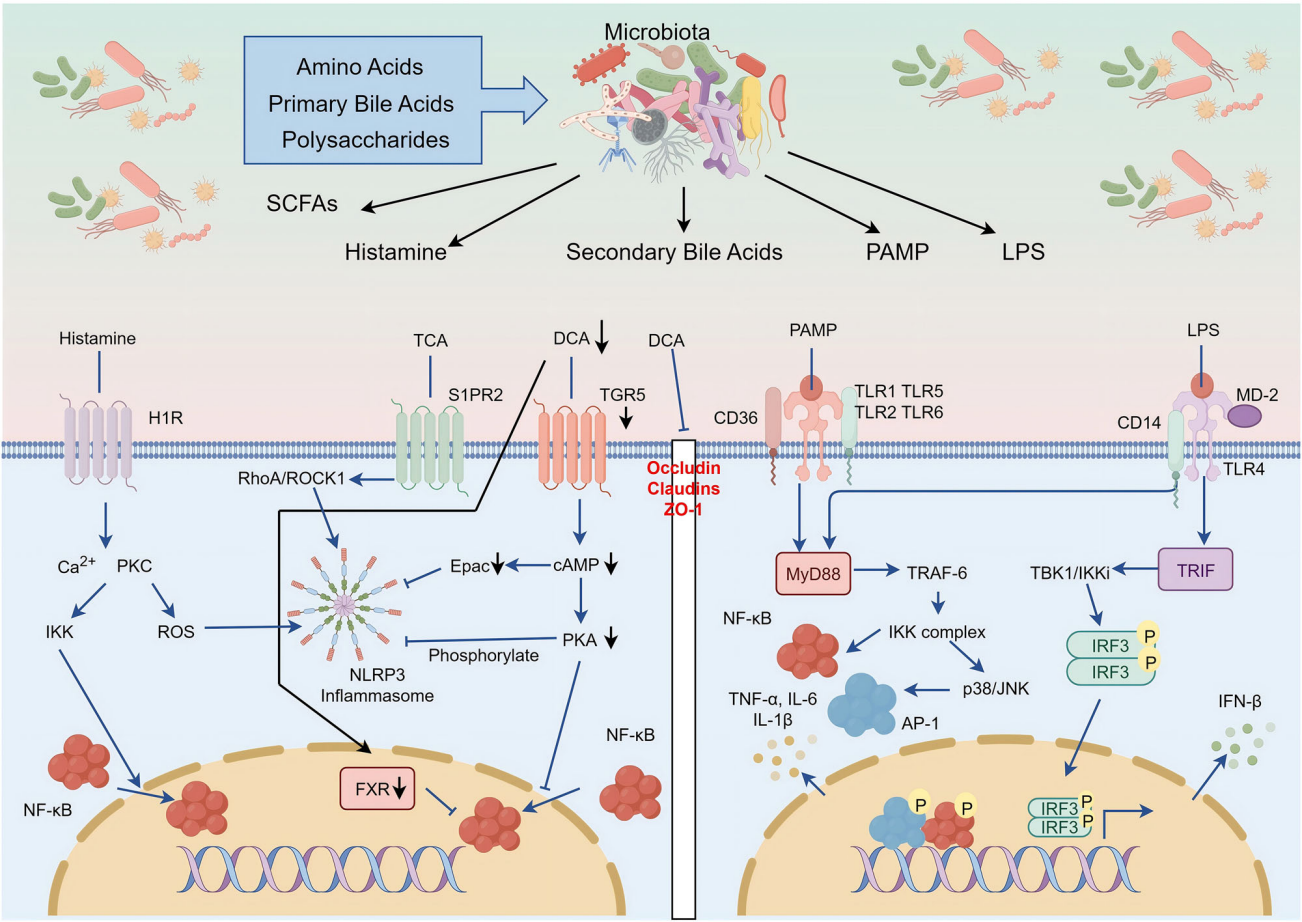
Principal molecular mechanisms underlying inter-organ axis crosstalk and systemic integration under pathological conditions. This schematic illustrates how intestinal dysbiosis drives systemic inflammation through multiple signaling pathways. Under disease states, microbiota alterations lead to the accumulation of histamine, LPS, and PAMPs, alongside a shift in the bile acid profile. Elevated histamine activates the H1R-Ca²⁺-PKC-IKK axis, triggering NF-κB nuclear translocation. LPS and other PAMPs engage TLR4 and other TLRs, activating the MyD88-dependent pathway (leading to the activation of NF-κB, p38/JNK, and AP-1) and the TRIF-dependent pathway (involving TBK1/IKKi and IRF3). In the bile acid signaling branch, reduced DCA levels weaken the TGR5-cAMP-PKA/Epac inhibitory axis, thereby diminishing the suppression of the NLRP3 inflammasome and NF-κB, while also contributing to the downregulation of FXR. Concurrently, TCA promotes inflammatory responses via the S1PR2-RhoA/ROCK1 pathway. These converged signals drive the release of pro-inflammatory cytokines (TNF-α, IL-6, IL-1β) and disrupt tight junction proteins (Occludin, Claudins, and ZO-1), ultimately exacerbating tissue injury. AP-1 Activator protein 1, cAMP Cyclic adenosine monophosphate, CD14/CD36 Cluster of differentiation 14/36, DCA Deoxycholic acid, Epac Exchange protein directly activated by cAMP, FXR Farnesoid X receptor, H1R Histamine receptor 1, IFN-β Interferon-beta, IKK IκB kinase, IKKi IκB kinase-epsilon, IRF3 Interferon regulatory factor 3, JNK c-Jun N-terminal kinase, LPS Lipopolysaccharide, MyD88 Myeloid differentiation primary response 88, NF-κB Nuclear factor-kappa B, NLRP3 NOD-like receptor protein 3, PAMPs Pathogen-associated molecular patterns, PKA Protein kinase A, PKC Protein kinase C, RhoA/ROCK1 Ras homolog family member A/Rho-associated protein kinase 1, ROS Reactive oxygen species, S1PR2 Sphingosine-1-phosphate receptor 2, TBK1 TANK-binding kinase 1, TCA Taurocholic acid, TGR5 Takeda G protein-coupled receptor 5, TLR4 Toll-like receptor 4, TNF-α/IL-6/IL-1β Tumor necrosis factor-alpha/Interleukin-6/Interleukin-1 beta, TRIF TIR-domain-containing adapter-inducing interferon-β, ZO-1 Zonula occludens-1.

The systemic integration of the bovine holobiont is maintained by a diverse array of biochemical signals that bridge distant anatomical sites. When localized mucosal barriers—such as those in the rumen, intestine, or mammary gland—are compromised by physiological stress or dysbiosis, specific molecular mediators enter the peripheral circulation. These mediators, including microbial structural components and metabolic byproducts, act as “molecular messengers” that transduce local microbial activity into systemic physiological or pathological responses (Fig. 2).

### Pathogen-associated molecular patterns

PAMPs are highly conserved microbial structural components, such as LPS, lipoteichoic acid (LTA), and peptidoglycan (PGN). Upon entering the systemic circulation, PAMPs are recognized by host pattern recognition receptors, most notably TLRs, on distant immune and epithelial cells. This recognition initiates downstream signaling cascades, amplifying a localized microbial imbalance into a systemic inflammatory state^143^.

As one of the most conserved structures in Gram-negative bacteria, LPS is recognized by the host to initiate potent immune responses against bacterial invasion. Uncontrolled overgrowth of bacteria in dairy cows often leads to the massive release of soluble LPS, which over-amplifies systemic immune reactions. When intestinal barrier integrity is compromised or permeability increases, LPS enters the circulation via the paracellular pathway or through lipoprotein-mediated transcellular transport. Once in circulation, LPS primarily activates the MyD88/TRIF signaling pathways via the LBP–CD14–MD-2–TLR4 complex, inducing the release of pro-inflammatory cytokines facilitated by NF-κB and IRF3. Additionally, cytosolic LPS is directly sensed by inflammatory caspases (e.g., Caspase-4), triggering non-canonical inflammasome responses and pyroptosis, which further amplifies systemic inflammation^144^.

LTA is typically released into the systemic circulation upon bacterial lysis or mucosal barrier impairment. The core mechanism of LTA-induced inflammation involves recognition by the TLR2/TLR6 heterodimer, a process facilitated by the co-receptors CD14 and CD36. Following receptor binding, the MyD88-dependent signaling pathway is primarily activated, leading to the robust stimulation of key signaling cascades such as NF-κB and MAPKs (e.g., p38 and JNK). This activation governs the gene transcription and synthesis of pro-inflammatory cytokines, including TNF-α, IL-6, and IL-1β. Additionally, the PI3K-Akt pathway is often synergistically activated, modulating cell survival, metabolism, and inflammatory signaling during the immune response^145^.

Furthermore, PGN is primarily recognized by intracellular NOD1 and NOD2 receptors, which activate the NF-κB and MAPK signaling pathways. Beyond intracellular sensing via NODs, PGN-derived fragments function as potent signals that trigger the assembly of the NOD-like receptor protein 3 (NLRP3) inflammasome, leading to the maturation and secretion of IL-1β. While the direct recognition of PGN by TLR2 remains a subject of academic debate, its synergistic interplay with other TLR-mediated pathways is critical for orchestrating the robust inflammatory response characteristic of bovine systemic dysbiosis. This multi-receptor recognition mechanism enables PGN to exert significant synergistic effects with other PAMPs, such as LPS, thereby amplifying systemic inflammation during inter-organ axes pathogenesis^146^.

### Bile acids

BAs act as pleiotropic signaling molecules that regulate intestinal homeostasis by integrating metabolic, epithelial, and immune pathways through receptor-mediated mechanisms. Their biological effects are highly dependent on BA composition and receptor context, resulting in context-dependent regulatory outcomes.

The anti-inflammatory pathways are primarily mediated by the farnesoid X receptor (FXR) and the Takeda G protein-coupled receptor 5 (TGR5)^147^. As a primary BA sensor, the canonical function of FXR is to maintain the BA pool homeostasis by regulating its synthesis, transport, and secretion. Beyond this physiological role, FXR serves as a critical bridge between metabolism and immunity. Activation of FXR in dendritic cells, macrophages, and natural killer T (NKT) cells suppresses the NF-κB and NLRP3 inflammasome pathways, thereby attenuating the secretion of pro-inflammatory cytokines (e.g., TNF-α, IL-6, and IL-1β) while promoting the release of the anti-inflammatory mediator IL-10. Similarly, TGR5 activation elevates intracellular cAMP levels, leading to downstream activation of protein kinase A (PKA) and exchange protein directly activated by cAMP (Epac), which suppresses NF-κB signaling, inhibits NLRP3 inflammasome activation, and can promote macrophage polarization from the pro-inflammatory M1 phenotype toward the anti-inflammatory M2 phenotype^148^.

Conversely, BAs can trigger pro-inflammatory cascades, primarily through sphingosine-1-phosphate receptor 2 (S1PR2)^149^. Activation of S1PR2 by specific BAs, particularly conjugated BAs such as taurocholic acid (TCA), stimulates the RhoA/ROCK1 signaling axis in macrophages, facilitating M1 polarization and NLRP3 inflammasome activation. In neutrophils, S1PR2 signaling enhances migration, promotes the formation of neutrophil extracellular traps (NETs), and extends cell survival, thereby exacerbating inflammatory injury^150^.

Beyond direct immune signaling, BAs significantly influence systemic inflammation by modulating intestinal permeability. Hydrophobic BAs, such as chenodeoxycholic acid (CDCA) and deoxycholic acid (DCA), have been shown to impair epithelial barrier function by disrupting the expression and structural integrity of tight junction proteins, including Occludin, ZO-1, and Claudins^151^. This barrier disruption can contribute to the translocation of luminal PAMPs into the systemic circulation, further amplifying inflammatory responses across the inter-organ axes.

### Histamine

Histamine, a potent biogenic amine, serves as a pivotal chemical messenger linking gastrointestinal dysfunction to peripheral pathologies, particularly laminitis, in dairy cows. Under normal physiological conditions, intestinal histamine is maintained at low levels through degradation by diamine oxidase (DAO) and histamine N-methyltransferase (HNMT). However, abnormal microbial fermentation, characterized by the proliferation of histamine-producing bacteria such as *Allisonella histaminiformans*, leads to substantial accumulation. Histamine exerts pleiotropic biological effects by activating four specific receptors (H1R–H4R) on tissue and vascular endothelial cells. Regarding immune regulation, histamine possesses significant chemotactic properties, inducing the migration of immune cells via H4R, while H2R mainly mediates immunosuppressive effects^152^. It also stimulates the release of pro-inflammatory cytokines, such as IL-6 and TNF-α, and may synergize with circulating LPS to exacerbate systemic inflammation. In terms of barrier function, high concentrations of histamine may disrupt intestinal epithelial tight junctions by activating H1R. This barrier damage not only results in the entry of histamine into the bloodstream, causing elevated systemic histamine levels, but also facilitates the transepithelial translocation of other PAMPs^153^. Consequently, this induces a vicious cycle across multiple organ axes and continuously amplifies the inflammatory response.

### Extracellular vesicles

Extracellular vesicles (EVs) are membrane-bound phospholipid bilayer structures released by virtually all cellular organisms, including both prokaryotes and eukaryotes. By encapsulating a diverse repertoire of biomolecules—such as nucleic acids, lipids, metabolites, and proteins—EVs serve as critical mediators of intercellular communication and host-microbe interactions. Furthermore, extensive research has demonstrated that EVs can also carry antigens, toxins, and pro-inflammatory cytokines, potentially serving as drivers of inter-organ diseases^154–156^.

The capacity of EVs to mediate inter-organ communication depends on their ability to traverse biological barriers via paracellular or transcellular pathways. While paracellular transmigration involves passage through intercellular junctions—often exacerbated by disrupted tight junctions under pathological conditions—transcellular transport is facilitated by various endocytic mechanisms, such as clathrin-mediated endocytosis, macropinocytosis, and lipid raft-dependent pathways^157–161^. Furthermore, bacterial-derived EVs (BEVs) can be specifically recognized and internalized by phagocytic cells through pattern-recognition receptors responding to surface components like LPS or LTA^162^.

After endocytosis, most EVs are transported to lysosomes for degradation. However, a subset of EVs can evade degradative pathways and enter recycling or transcytotic routes^157,163^. Once in circulation, EVs may form complexes with lipoproteins to enhance their stability and delivery to distant organs^164^. In dairy cows, BEV-mediated transport of LPS and virulence factors during dysbiosis—such as in SARA or mastitis—represents a plausible but underexplored mechanism linking localized microbial imbalance to systemic inter-organ pathology.

### Short-chain fatty acids and tryptophan metabolites

SCFAs, including acetate, propionate, and butyrate, represent the dominant end-products of ruminal and hindgut microbial fermentation. Beyond their established role as energy substrates, SCFAs act as localized and systemic signaling molecules through G protein-coupled receptors (GPR41, GPR43, and GPR109A), thereby mediating bidirectional communication between the gastrointestinal tract and peripheral organs^165^. For instance, in SARA goats, supplementation with sodium butyrate significantly reduced the translocation of LPS from the rumen into the peripheral blood. Notably, this reduction in systemic LPS was accompanied by a down-regulation of pro-inflammatory mediators (e.g., TLR4, NF-κB, and IL-6) in the uterus, suggesting that butyrate can alleviate high-grain induced inflammation across the gut-uterus axis by fortifying the epithelial barrier^166^.

In addition to SCFAs, microbial tryptophan metabolites represent another class of important signaling molecules. Intestinal microorganisms can convert dietary tryptophan into a variety of indole derivatives, including indole, indole-3-acetic acid, indole-3-propionic acid, and indole-3-aldehyde. These metabolites function as ligands for host receptors such as the aryl hydrocarbon receptor (AhR) and pregnane X receptor (PXR), thereby regulating epithelial barrier integrity, mucosal immunity, and systemic inflammatory responses^167^. For example, in ketotic dairy cows, the ruminal level of the tryptophan metabolite indole-3-acetic acid (IAA) is significantly reduced and is accompanied by downregulation of epithelial tight junction proteins, increased inflammatory mediators, and impaired barrier function. Further in vitro studies have shown that IAA can promote epithelial cell proliferation and enhance tight junction repair by activating the AhR/IL-22 signaling pathway, thereby counteracting LPS-induced epithelial injury and preventing LPS-driven systemic inflammation^168^.

## Gut-mediated inter-organ axes

### Gut–liver axis: a central hub of inter-organ crosstalk

**Fig. 3:**
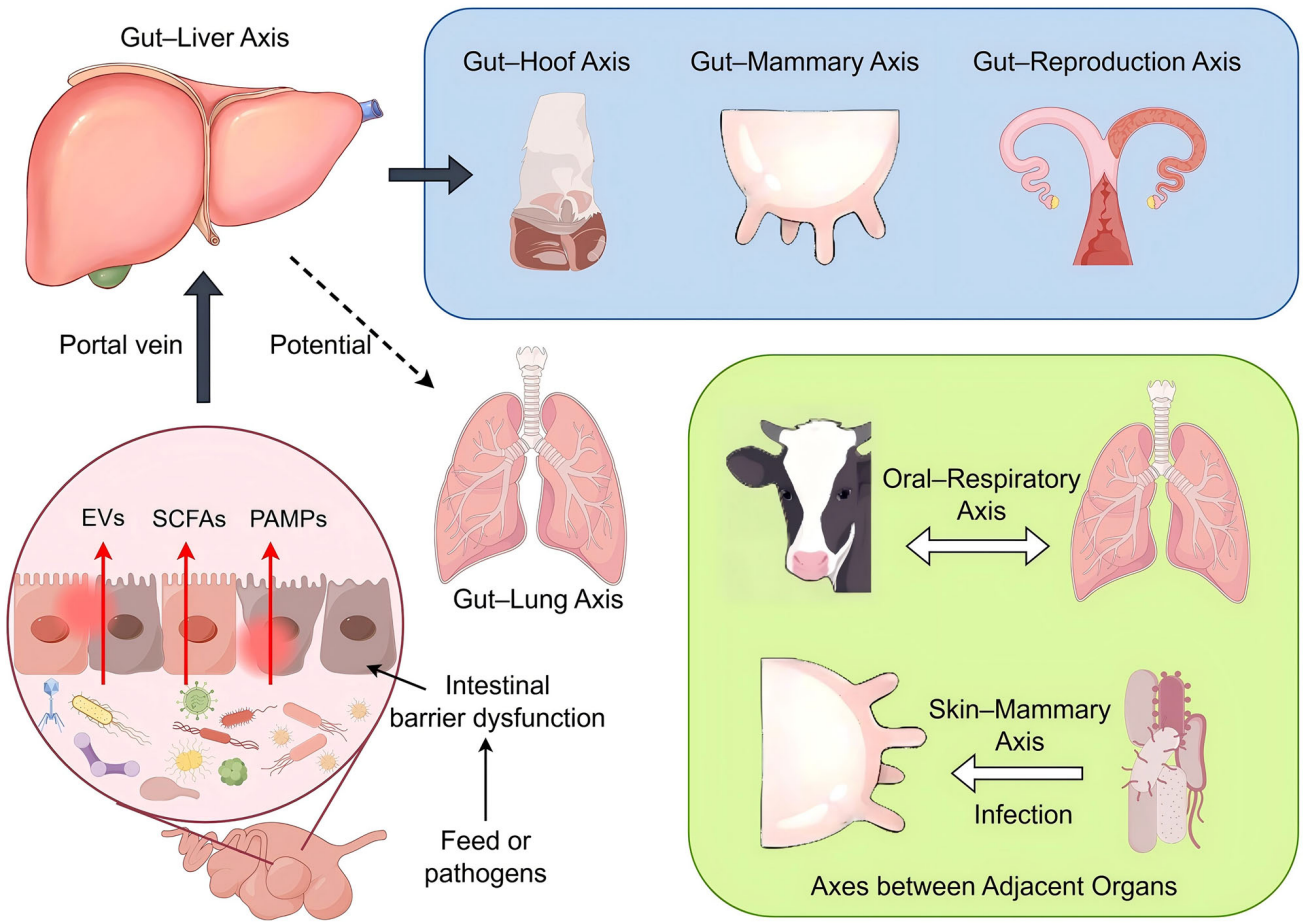
Schematic overview of inter-organ axes in dairy cows. Microbial dysbiosis induced by diet or pathogen invasion can impair intestinal barrier function, leading to the release of microbiota-derived signals into the portal circulation. This process positions the gut–liver axis as a hub for inter-organ communication. Downstream systemic axes—including the gut–hoof axis, gut–mammary axis, and gut–reproductive axis—receive dysbiosis-derived signals through hepatic processing and systemic circulation. However, the functional involvement of the liver in the bovine gut–lung axis remains poorly supported by current evidence. In contrast, local microbiome axes between adjacent organs, such as the oral–respiratory axis and the skin–mammary axis, operate through direct anatomical continuity rather than hematogenous dissemination. EVs extracellular vesicles, SCFAs short-chain fatty acids, PAMPs pathogen-associated molecular patterns.

The gastrointestinal tract and the liver constitute two major metabolic organs in dairy cows and are functionally interconnected through a bidirectional gut–liver axis mediated by multiple signaling molecules. Beyond its intrinsic metabolic functions, the gut–liver axis also acts as a central hub of inter-organ communication (Fig. 3). A substantial proportion of microbiota-derived components and metabolites from the gastrointestinal tract are transported to the liver through the portal vein. When these signals exceed the detoxification capacity of the liver, they may spill over into the systemic circulation, thereby propagating pathological cascades to peripheral organs. Notably, in ruminants, the ruminal microbiota represents a central and distinctive component of the gut–liver axis.

As previously described, perinatal dietary transitions often induce ruminal dysbiosis, which compromises glucogenic precursor supply and promotes extensive fat mobilization, thereby leading to excessive ketone body production in the liver. Research utilizing mediation analysis models confirmed that key genera, including *Prevotella* and *UBA1066*, influence serum BHBA and glucose concentrations by regulating propionate and glucogenic amino acid levels. Furthermore, the observation that sodium propionate supplementation effectively reduces serum ketone levels in ketotic cows validates the mediating role of rumen-derived propionate in the gut–liver axis and ketosis^47^. Dysbiosis of the hindgut microbiota, characterized by the depletion of acetate-producing genera (e.g., *Faecousia*) and the expansion of acetate-consuming methanogens (e.g., *Methanosphaera*), leads to a deficiency in intestinal acetate. This deficiency signals via the gut–liver axis to suppress the hepatic AMPK–PPARA signaling pathway, thereby impairing fatty acid oxidation and exacerbating lipid accumulation in the liver. Consequently, these metabolic shifts promote ketogenesis and the development of ketosis. In vitro experiments using primary hepatocytes confirm that acetate supplementation reverses these effects by activating the AMPK–PPARA axis, underscoring the critical role of microbial-derived acetate in regulating hepatic energy homeostasis^169^.

The pathogenesis of SARA further illustrates the dynamic crosstalk within the gut–liver axis. Abnormal microbial fermentation induces the lysis of Gram-negative bacteria and the subsequent release of microbial metabolites, including LPS, histamine, iE-DAP, and muramyl dipeptide (MDP)^170^. These metabolites translocate across the compromised ruminal epithelial barrier into the systemic circulation, reaching the liver via the portal vein and subsequently triggering multiple injury mechanisms, as previously described^170^. Simultaneously, LPS significantly inhibits the expression of hepatic SIRT1 and FOXA2, which downregulates the NFE2L2/HMOX1 antioxidant signaling axis, diminishes the activity of key antioxidant enzymes (e.g., CAT, SOD, and GPX1), and upregulates autophagy-related proteins such as MAP1LC3-II and ATG5^171^. This culminates in a combined insult of oxidative stress, excessive autophagy, and inflammatory damage in the liver.

### Gut–hoof axis

Gut-derived endotoxins and pro-inflammatory mediators can spill into the systemic circulation, inducing persistent low-grade inflammation. This state functionally extends the gut–liver axis to peripheral tissues, thereby giving rise to additional inter-organ communication axes, such as the gut–liver–hoof axis^172^. Histological analyses have demonstrated that high-starch diets inducing SARA reduce both the length and width of hoof epidermal lamellae in dairy cows^173^. Within this framework, LPS and histamine exert a critical priming effect: circulating LPS lowers the inflammatory activation threshold of peripheral tissues, while rumen-derived histamine promotes microvascular dysregulation and lamellar hypoxia. Although peak inflammatory cytokine production (e.g., IL-1β) may stem from localized MAPK/ERK dysregulation rather than direct endotoxin influx^174^, the cumulative impact of gut–liver axis dysfunction in destabilizing cellular homeostasis remains an indispensable driver of the systemic pathogenesis of bovine laminitis.

### Gut–lung axis

Respiratory diseases represent one of the primary threats to the health of dairy cattle, particularly young calves, and accumulating evidence supports a functional gut–lung axis in this context. The regulatory influence of the gut microbiota on pulmonary immune homeostasis has been demonstrated through microbiota transfer (MT) experiments. Combined RMT and FMT reduced plasma neutrophil concentrations and significantly decreased TNF-α in BALF, confirming that gastrointestinal microbiota composition can reshape the pulmonary cytokine profile. However, this anti-inflammatory effect did not translate into improved clinical outcomes, as clinical scores increased and antimicrobial usage rose following cessation of MT^175^, suggesting that the gut–lung axis involves complex mechanisms that require further optimization.

Compared with MT, dietary supplementation may represent a more practical approach to modulating the gut**–**lung axis to support calf health. Oral supplementation with non-digestible oligosaccharides (NDO), such as galacto-oligosaccharides (GOS) and fructo-oligosaccharides (FOS), can mitigate both systemic and respiratory inflammation. These prebiotics reshape the profile of circulating inflammatory mediators, leading to a significant reduction in pro-inflammatory cytokines—including TNF-α, IL-6, IL-8, and IL-1β—within both plasma and BALF^176^. In calves experimentally coinfected with BRSV and *Pasteurella multocida*, feeding with *Saccharomyces cerevisiae* fermentation products (SCFP) attenuated pulmonary inflammatory responses and enhanced pulmonary plasminogen expression as well as the expression of genes involved in glutathione metabolism, indicating that gut-derived interventions can reshape systemic and pulmonary immunity^177^. Nevertheless, the precise mechanisms by which gut microbiota modulate pulmonary immunity remain to be fully elucidated; in particular, whether the gut**–**lung axis operates independently or is mediated in part through hepatic processing of gut-derived signals warrants further investigation.

### Gut–mammary axis

Beyond exogenous infection, endogenous mechanisms mediated by the “gut–mammary axis” have recently gained attention. FMT experiments have preliminarily confirmed a causal link between gut dysbiosis and mammary inflammation^135^. In dairy cow studies, researchers have also found that SARA induces mammary inflammatory symptoms, activates systemic inflammatory responses, and increases the permeability of the blood–milk, intestinal, and ruminal barriers. Further evidence indicates that LPS produced in the intestines of SARA cows can translocate into the bloodstream, impair hepatic function, and subsequently accumulate in the mammary gland through the gut–liver–mammary axis, triggering TLR4–NF-κB–NLRP3-mediated inflammatory responses^178^. Beyond direct LPS translocation, SARA-induced intestinal accumulation of free sialic acid further downregulates tight junction proteins and amplifies mucosal permeability, reinforcing this pathological cascade^179^. Beyond LPS translocation, pathogenic bacteria may also migrate directly through the gut–mammary axis and contribute to mastitis development. Experimental evidence from murine models has shown that, following oral administration of green fluorescent protein (GFP-labeled *Escherichia coli*), a detectable GFP signal can be observed in mammary tissue. However, whether such bacterial translocation occurs in dairy cows remains to be established^180^. *Staphylococcus aureus* mastitis isolates produce EVs containing α-haemolysin, which, upon stimulation of bovine mammary epithelial cells in vitro, induce TNF-α upregulation, demonstrating that pathogen-derived EVs can directly modulate mammary epithelial immune responses independent of systemic gut-derived signals. Whether EVs originating from gut dysbiosis can similarly reach and prime the mammary gland through the gut–mammary axis, however, remains to be investigated^181^.

While the mechanisms discussed above primarily describe how gut dysbiosis drives pro-inflammatory signals toward the mammary gland, the gut–mammary axis also operates through immunoprotective pathways. In murine models, gut dysbiosis impaired the differentiation of IgA⁺ B cells into plasma cells, reducing milk sIgA levels and weakening the mammary mucosal immune barrier, thereby exacerbating *S. aureus*-induced mastitis and blood–milk barrier disruption. Mechanistically, beneficial microbes such as *Akkermansia* synthesize GABA, which activates the mTOR signaling pathway to promote both M2 macrophage polarization and plasma cell differentiation, sustaining sIgA production and mammary mucosal defense^182^. Although these findings are primarily derived from murine models, they highlight the dual nature of the gut–mammary axis as both a conduit for pathological signals and a mediator of mucosal immune defense, warranting further investigation in dairy cows.

### Gut–reproduction axis

The gastrointestinal tract significantly influences reproductive health through the gut–reproduction axis, which operates in part as a downstream extension of gut–liver axis dysfunction. Direct evidence for this gut–liver–reproduction axis comes from a study in dairy goats with SARA, in which high-concentrate diet-induced ruminal dysbiosis was accompanied by elevated endometrial LPS levels, increased neutrophil infiltration, and disruption of uterine tight junction proteins^183^. Beyond LPS, gut-derived components can also translocate through the bloodstream along this axis, a process that may exert a double-edged effect on uterine health. On the pathological side, intestinal dysbiosis and compromised barrier integrity may allow pathogenic bacteria (e.g., *Fusobacterium*) to enter the systemic circulation. These mediators subsequently reach the uterus, where they trigger local inflammatory cascades that impair uterine recovery and promote the development of bovine metritis. This hypothesis is supported by the detection of such pathogens in peripheral blood during the periparturient period^129^. Conversely, the gut–reproduction axis also serves as a vital conduit for protective signaling. Emerging evidence from murine models indicates that maternal gut microbiota supports placental development through SCFAs, since depletion of the maternal gut microbiota impairs feto-placental vascularization, whereas SCFA supplementation restores placental growth and vascular integrity^184^. Collectively, these findings suggest that the gut–reproduction axis is not merely a route for endogenous infection but rather a complex regulatory pathway that bidirectionally influences reproductive health. Recent studies in mammals have further demonstrated that the gut microbiota can influence gonadal function through multiple mechanisms, including the regulation of circulating steroid hormones, insulin sensitivity, and immune homeostasis^185^. These observations have led to the proposal of a gut–gonadal axis, which may represent a future research direction in dairy cattle.

## Local microbial axes between adjacent organs

The inter-organ axes described above operate primarily through systemic dissemination of microbial-derived signals via the portal circulation and peripheral bloodstream, with the gut–liver axis serving as the central hub. However, in anatomically adjacent organ systems, direct microbial translocation through physical continuity represents an alternative and underappreciated axis of inter-organ crosstalk.

### Skin–mammary axis

The stability of the mammary gland is closely associated with udder health and milk quality. Microbes are generally believed to enter the mammary gland by breaching the physical barrier of the teat canal. Physiological thinning of the teat canal keratin layer before parturition and transient dilation of the teat sphincter after milking are considered key windows for pathogen invasion^186^. Both culture-based and sequencing studies have identified a shared core microbiota across teat skin, teat canal, and mammary gland cistern, dominated by non-*aureus Staphylococci* (NAS) and *Corynebacterium*, and including some environment-associated taxa such as Clostridiaceae and *Acinetobacter*^187^, providing microbial evidence for a continuous skin–mammary axis.

The clinical relevance of this axis is further supported by epidemiological evidence: a prospective cohort study demonstrated that teat skin condition was directly associated with clinical mastitis occurrence, with combined dry skin and skin lesions conferring nearly five-fold higher odds of clinical mastitis compared with normal teat skin^188^, underscoring teat skin integrity as a critical determinant of mammary health along this axis. A longitudinal study tracking microbial communities in bedding, teat skin, and milk further supported environmental-to-mammary transmission along the skin–mammary axis. With prolonged bedding use, the abundance of pathogenic bacteria in milk increased, accompanied by a rise in somatic cell counts, indicating that environmental microbial shifts may influence mammary health via sequential colonization of the teat skin and mammary gland^189^. The skin–mammary axis may also represent a feasible intervention target. The addition of bacterial modulators to recycled manure solid bedding significantly reduced the abundance of potential mastitis pathogens on teat skin in cows housed on treated bedding, accompanied by improvements in milk somatic cell counts^190^.

However, the relationship within the skin–mammary axis is not always straightforward. A study investigating teat-end hyperkeratosis in commercial dairy herds found no significant association between hyperkeratosis and mastitis-causing pathogens^191^. These apparently conflicting findings suggest that interactions along the skin–mammary axis are likely modulated by additional factors, such as milking management, environmental hygiene, and host immune status.

### Oral–respiratory axis

Although oral microorganisms can also influence respiratory health through systemic routes—including LPS dissemination, outer membrane vesicles, and pro-inflammatory cytokines entering the circulation—the defining feature of the oral–respiratory axis is the direct physical translocation of oral microbiota into the lower airways via microaspiration^192^. In ruminants, this axis may extend to incorporate the rumen as a microbial reservoir: the unique physiology of rumination and eructation creates a cyclical “rumen**–**oral**–**lung” conduit through which rumen-associated taxa, including *Prevotella*, *Ruminococcus*, and *Clostridium butyricum*, may periodically enter the respiratory tract, as evidenced by their detection in bovine lung samples^193,194^. Within the framework of the oral**–**respiratory axis, metabolites produced by oral microorganisms may also act as signaling molecules. Oral anaerobes generate volatile sulfur compounds (VSCs) such as hydrogen sulfide and methanethiol, which may modulate inflammatory signaling and epithelial integrity within the respiratory mucosa^195^, while organic acids and tryptophan-derived metabolites produced by oral commensals may further alter microbial colonisation patterns and host immune responses in the airways^196^. Given that orally administered prebiotics and postbiotics have been shown to improve pulmonary health in calves—potentially through modulation of the gut**–**lung axis—the oral microbiome, as a nodal point within this axis, represents an accessible intervention point warranting further investigation in bovine-specific models.

## Emerging microbiome-based strategies in dairy cow: opportunities and challenges

**Fig. 4:**
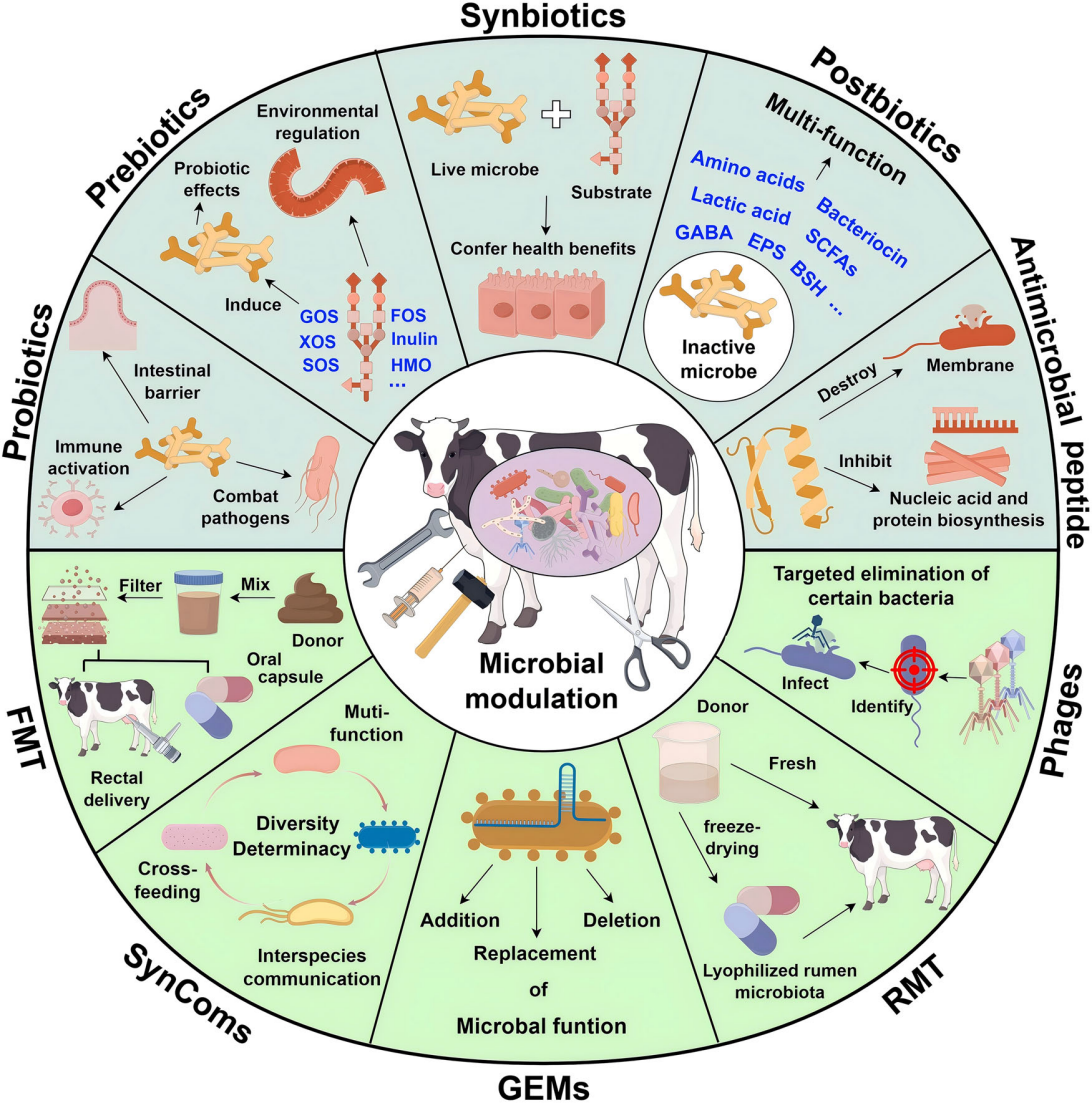
Microbiome-based intervention strategies for system-level modulation of dairy cow health. Conventional approaches, including probiotics, prebiotics, synbiotics, postbiotics, and antimicrobial peptides, are shown in the upper sector and primarily act through indirect regulation of microbial growth, metabolic activity, or host responses. These strategies are generally safe and scalable but often provide limited control over community-level functions and microbiota–organ axis stability, particularly under conditions of severe dysbiosis or high metabolic stress. In contrast, the lower sector highlights next-generation microbiome engineering strategies, including fecal microbiota transplantation, rumen microbiota transplantation, synthetic microbial communities, phages, and genetically engineered microorganisms. These approaches enable more targeted and systematic manipulation of microbial consortia, functional networks, or specific microbial signals through donor-derived community replacement, early-life microbial programming, selective bacterial elimination, or precise genetic and metabolic editing. Collectively, these emerging strategies represent a shift from indirect nutritional modulation toward system-level control of microbiota–organ axes, offering new opportunities to enhance metabolic homeostasis, immune resilience, and long-term productivity in dairy cattle. FMT Fecal microbiota transplantation, SynComs Synthetic microbial communities, GEMs Genetically engineered microbes, RMT Rumen microbiota transplantation, GOS/FOS/XOS/SOS Galacto/Fructo/Xylo/Soy-oligosaccharides, HMO Human milk oligosaccharides, SCFAs Short-chain fatty acids, BSH Bile salt hydrolase, EPS Exopolysaccharides, GABA Gamma-aminobutyric acid.

Growing evidence from microbiota–organ axis studies indicates that dysbiosis-related disorders in dairy cows are driven by coordinated disturbances in microbial community structure and function across multiple organs, rather than by changes in individual taxa or metabolites. Consequently, effective microbiome modulation requires intervention strategies capable of reshaping microbial consortia and functional networks at a system level. In this context, emerging approaches such as FMT, RMT, SynComs, phages, and GEMs have attracted increasing attention for their potential to restore microbiota–organ axis homeostasis and enhance dairy health (Fig. 4). While emerging strategies such as FMT, RMT, SynComs, phages, and GEMs are the primary focus of this section, approaches including probiotics, prebiotics, synbiotics, postbiotics, and antimicrobial peptides remain integral components of microbiome management in dairy cattle (Table 3).

**Table 3:** Other in vivo studies of microbiome modulation strategies in dairy cattle

Strategy	Components	Subject	Outcome	Reference
Probiotics	Multi-strain probiotic: *Lactobacillus*, *Enterococcus faecium*, *Saccharomyces cerevisiae* & *Bacillus licheniformis*	Newborn Holstein calves	ADG ↑ 43.40%; diarrhea rate ↓42.90%; enhanced immunity	Cai et al.^242^
	Two probiotic formulas composed of multiple *Lactobacillus*, *Bifidobacterium*, *Enterococcus* & *Bacillus*, delivered via milk or cassava carrier	Newborn Holstein calves	No effect on intestinal disease incidence or pre-weaned heifer growth	Velasquez-Munoz et al.^243^
	Multi-strain probiotic: *Clostridium beijerinckii*, *Pichia kudriavzevii*, *Ruminococcus bovis* & *Butyrivibrio fibrisolvens*	Multiparous & primiparous Holstein cows	Milk yield ↑; feed conversion ratio ↑	Bulnes et al.^244^
	Live *Saccharomyces cerevisiae*	SARA Holstein cows	Attenuated SARA	AlZahal et al.^245^
	Intravaginal infusion of Lactic Acid Bacteria cocktail	Pregnant Holstein cows	Reduced incidence of metritis	Deng et al.^246^
	Intrauterine infusion of *Lactobacillus buchneri* DSM 32407	Holstein cows with subclinical endometritis	Improved reproductive performance	Peter et al.^247^
	Intranasal administration of six *Lactobacillus* strains	Holstein bull calves	Reduced Mannheimia haemolytica colonization in calf nasopharynx	Amat et al.^248^
Prebiotics	FOS	Newborn Holstein calves	↑ ADG	Gao et al.^249^
	GOS	Holstein bull calves	Promoted intestinal development, despite mild diarrhea	Castro et al.^250^
	GOS and FOS	Holstein bull calves	No synergistic effect observed for co-supplementation; individual addition was more effective	Gilbert et al.^176^
	GOS	Calves with lung infections	Anti-inflammatory effect via inhibition of NLRP3 inflammasome activation	Cai et al.^251^
	Inulin	Dairy cows with subclinical mastitis	300 g/d inulin supplementation attenuated inflammation	Wang et al.^252^
Synbiotics	MOS + *Bacillus subtilis*	Newborn Holstein calves	From d 42 to 56, ADG of synbiotic-treated calves was 78 g higher than the control	Lucey et al.^253^
	FOS + *Lactobacillus plantarum* CRD-7	Buffalo calves	Better performance in digestibility, ADG, antioxidant enzymes, immunity	Sharma et al.^254^
Postbiotics	SCFP	Multiparous Holstein cows	Regulated immunity & hepatic metabolism; promoted ECM yield; improved inflammatory status	Dai et al.^255^
	SCFP	Holstein calves infected with BRSV and Pasteurella multocida	Regulated systemic & mucosal immune responses; attenuated pulmonary inflammation	Maina et al.^177^
Antibiotics	Hetacillin potassium	Dairy cows with clinical mastitis	68% cure rate	Vasquez et al.^256^
	Cloxacillin benzathine + internal teat sealant	Dry cows	Reduced prevalence of Gram+ microbes infections	Peña-Mosca et al.^257^
	Intramuscular ceftiofur	Holstein cows with metritis	Increased abundance of antibiotic-resistant bacteria	Vasco et al.^258^
AMPs	Bacteriocin MccJ25 + Chinese herbal formula	Diarrheic calves	Combination therapy effectively reduced diarrhea incidence in healthy calves in a dose-dependent manner	Ding et al.^259^
	AMP + Tributyrin	Heifer calves	Individual addition of AMP increased ADG and weaning weight and reduced diarrhea; combined use did harm to calf performance	Gao et al.^260^

*ADG* Average Daily Gain, *SARA* Subacute Ruminal Acidosis, *FOS* Fructooligosaccharides, *GOS* Galactooligosaccharides, *MOS* Mannanoligosaccharides, *SCFP* *Saccharomyces cerevisiae* Fermentation Products, *BRSV* Bovine Respiratory Syncytial Virus, *AMPs* Antimicrobial Peptides, *ECM* Energy-Corrected Milk, *NLRP3* NOD-like Receptor Protein 3.

### Fecal and rumen microbiota transplantation

Microbial transplantation, encompassing FMT and RMT, is a holistic strategy designed to restore ecosystem stability by transferring complex microbial communities from healthy donors to recipients. Its application in ruminants has yielded significant outcomes. In a landmark study involving 57 diarrheic beef calves, FMT was shown to markedly alleviate clinical symptoms. A longitudinal multi-omics analysis indicated that the therapeutic efficacy of FMT is associated with the enrichment of Porphyromonadaceae. Notably, a 24-month follow-up confirmed that these benefits extend beyond acute recovery, exerting sustained positive effects on subsequent growth performance^197^.

In lactating cows, RMT has also been explored for the treatment of ruminal metabolic disorders. Studies on SARA have shown that cows receiving RMT from healthy donors recovered acetate, valerate, and total VFA concentrations more rapidly and exhibited a significant increase in rumen bacterial community diversity. Without disrupting the core microbial network that maintains rumen functional stability, RMT promoted the growth of fiber-degrading taxa such as Ruminococcaceae and *Saccharofermentans*, thereby accelerating the recovery of fermentation function and ruminal homeostasis^51^. Beyond improving gut health, FMT has also shown potential in the treatment of extraintestinal diseases. A study involving 180 calves demonstrated that FMT reduced the proportion of neutrophils in plasma and decreased lymphocyte counts and TNF-α levels in bronchoalveolar lavage fluid, indicating attenuation of respiratory and systemic inflammation^175^.

Although FMT and RMT show promise in modulating gut microbiota and treating diseases in dairy cattle, several critical hurdles still stand in the way of their widespread practical application. First, donor screening is time-consuming and costly. Prior to microbiota transplantation, donors must undergo comprehensive veterinary health assessments, and the absence of pathogenic strains must be confirmed by culture, PCR, or sequencing. Failure to ensure donor safety may result in the transmission of antibiotic-resistant bacteria, harmful viruses, or proinflammatory metabolites^198^. Second, the success of FMT/RMT remains variable. Transplant outcomes depend not only on donor microbiota composition but also on the preexisting microbial structure of the recipient. Post-transplant microbial dynamics further reveal that different taxa exhibit distinct responses, including retention of original rhythmicity, altered or newly acquired rhythms, or loss of rhythmic patterns, highlighting the inherent uncertainty in microbial reconstruction following RMT^199^. In addition, cost remains a major challenge for large-scale applications. The operational procedures for FMT and RMT are labor-intensive, while post-transplant outcomes retain a degree of unpredictability. Their economic benefits in commercial dairy operations, therefore, still warrant careful consideration.

### Phages

Following chemical interventions such as antibiotics and AMPs, phage therapy provides a more targeted biological strategy. Several decades ago, phages were explored for the targeted control of pathogens to treat ruminant-associated diseases. For example, a single oral administration of the sheep-derived phage CEV1 was reported to reduce the abundance of *Escherichia coli* O157:H7 in the ovine intestine by approximately two log units within 2 days^200^. Golla et al. also constructed a library of lytic phages specific to MAP through isolation and culture. Some of these phages were found to prevent MAP colonization and invasion of bovine intestinal epithelial cells, indicating potential for prophylactic application^201^. Notably, the combined application of phages and probiotics may produce synergistic effects. A study on neonatal calf diarrhea showed that a formulation containing *Lactobacillus* spp. and *Escherichia coli*-specific phages not only completely resolved diarrhea symptoms within 24–48 h but also significantly enhanced both specific and nonspecific host immune responses^202^.

For extra-intestinal sites, the novel phage EF-N13 has demonstrated effective in vitro lysis of multidrug-resistant *Enterococcus faecalis* isolated from mastitic milk, while also inhibiting biofilm formation^203^. Phage cocktail strategies can further mitigate the risk of resistance while enhancing therapeutic efficacy. For *Staphylococcus aureus*-induced mastitis, researchers developed a three-phage cocktail PHC-1, which exhibited superior bactericidal activity and a lower frequency of resistant mutations compared to individual phages. When evaluated in a lactating mouse model, PHC-1 treatment reduced mammary bacterial loads and inflammatory responses, suggesting potential antimicrobial activity that requires validation in bovine systems^204^. However, phage efficacy may be environment-dependent. A large-scale trial (*n* = 597) revealed that intrauterine infusion of a four-phage cocktail failed to reduce *Escherichia coli* and *Trueperella pyogenes* detection in dairy cows, nor did it improve postpartum uterine health or reproductive performance^205^. Thus, bovine phage therapies require further refinement, specifically tailoring administration routes and bacterial host targets to the unique physiological environment of each disease.

### Synthetic microbial communities

SynComs consist of multiple microorganisms that can exert beneficial effects on the host. Unlike FMT/RMT, SynComs represent defined but incomplete microbial communities and are therefore theoretically associated with higher safety, controllability, and reproducibility^198^. In livestock research, early applications of the SynCom concept were mainly reflected in the combined use of multiple probiotics. For example, a study in neonatal calves administered a mixture of *Lactobacillus plantarum*, *Pediococcus acidilactici*, *Pediococcus pentosaceus*, and *Bacillus subtilis*. Although effects on body weight and average daily gain were limited, high-dose treatment significantly increased serum total protein, immunoglobulin levels, and superoxide dismutase activity, promoted ruminal butyrate production, and reduced diarrhea frequency^206^.

Recent studies have demonstrated the therapeutic potential of SynCom design for combating enteric pathogens. Zhang et al. constructed a 10-member synthetic consortium (SynComBac10) representing the core diversity of the adult chicken gut microbiota. When administered to newly hatched chicks, SynComBac10 promoted early colonization of segmented filamentous bacteria, inducing Th17 cell-mediated mucosal immunity and conferring significant resistance to *Salmonella* challenge, as evidenced by reduced bacterial loads, attenuated systemic inflammation, and improved intestinal barrier integrity. These results indicate that functionally defined SynCom can confer active anti-infective capacity through microbiota–immune axis modulation. Although direct validation in bovine systems remains necessary, the higher microbiota plasticity of neonatal calves compared with adult dairy cows suggests that SynCom-based interventions may hold promise for enteric disease management in early-life cattle^207^.

### Genetically engineered microorganisms

GEMs emphasize precise genetic and metabolic engineering at the strain level. This strategy focuses on accurate editing and optimization of intrinsic genetic systems rather than relying on plasmid-based expression systems, providing greater certainty, stability, and directionality^208^. In livestock systems, however, the practical application of GEMs remains extremely limited. Most studies are currently restricted to proof-of-concept experiments demonstrating the feasibility of microbial engineering. For example, genetically modified *Lactobacillus* strains have been explored as mucosal antigen delivery platforms, demonstrating the capacity to elicit both local IgA and systemic immune responses in experimental livestock models^209^.

Despite these advances, the application of GEMs in dairy cattle faces substantial regulatory, biosafety, and consumer acceptance challenges inherent to food-producing animals. Current GEM research in ruminants should therefore be regarded primarily as a mechanistic tool rather than an immediately deployable intervention strategy, pending rigorous evaluation of ecological stability, horizontal gene transfer risks, and relevant regulatory frameworks.

## Concluding perspective

In essence, the microbial consortia across the bovine gastrointestinal tract, reproductive system, respiratory tract, mammary gland, and skin perform specialized ecological and metabolic functions tailored to their local environments. However, these compartments are far from isolated. Disruption of mucosal barriers—driven by nutritional stress, parturition-associated immunosuppression, or pathogen invasion—facilitates the systemic translocation of microbial-derived signals, such as PAMPs, BAs, and SCFAs. These mediators link regional dysbiosis to distal organ dysfunction, underpinning the inter-organ axes described throughout this review that redefine our understanding of disorders like mastitis, ketosis, and BRD.

Looking forward, several priorities emerge for advancing microbiome-informed dairy health management. First, longitudinal multi-omics studies integrating metagenomics, metabolomics, transcriptomics, and host immunophenotyping across multiple organs are essential to disentangle causality from association within inter-organ axes. Second, standardized sampling frameworks and contamination-aware analytical pipelines are urgently needed, particularly for low-biomass niches such as the lung, uterus, and mammary gland. Third, functional validation—through microbial transplantation, targeted microbial modulation, and metabolite intervention experiments—will be critical to translate correlative findings into actionable mechanisms. Beyond research priorities, future disease management should move beyond site-specific probiotics or antimicrobials toward systemic microbiome stabilization approaches, leveraging diet formulation, early-life microbial programming, and host–microbe co-adaptation.

In summary, viewing the dairy cow as an integrated holobiont provides a unifying conceptual framework for understanding how microbial ecosystems collectively regulate health and disease. By bridging localized microbial dysbiosis with systemic physiological outcomes, this perspective not only advances fundamental microbiome science but also offers a theoretical foundation for next-generation strategies aimed at improving animal welfare, productivity, and sustainability in modern dairy systems.
